# The AusTraits plant dictionary

**DOI:** 10.1038/s41597-024-03368-z

**Published:** 2024-05-25

**Authors:** Elizabeth H. Wenk, Hervé Sauquet, Rachael V. Gallagher, Rowan Brownlee, Carl Boettiger, David Coleman, Sophie Yang, Tony Auld, Russell Barrett, Timothy Brodribb, Brendan Choat, Lily Dun, David Ellsworth, Carl Gosper, Lydia Guja, Gregory J. Jordan, Tom Le Breton, Andrea Leigh, Patricia Lu-Irving, Belinda Medlyn, Rachael Nolan, Mark Ooi, Karen D. Sommerville, Peter Vesk, Matthew White, Ian J. Wright, Daniel S. Falster

**Affiliations:** 1 Evolution & Ecology Research Centre, University of New South Wales, Sydney, Australia; 2 National Herbarium of NSW, Botanic Gardens of Sydney, Mount Annan, NSW Australia; 3 Hawkesbury Institute for the Environment, Western Sydney University, Sydney, Australia; 4 Australian Research Data Commons, Caulfield East, Australia; 5 Department of Environmental Science, Policy, & Management, University of California, Berkeley, USA; 6 NSW Department of Planning and Environment, Parramatta, Australia; 7 University of Wollongong, Wollongong, Australia; 8 Centre for Ecosystem Science, University of New South Wales, Syndey, Australia; 9 School of Biological Sciences, University of Tasmania, Hobart, Australia; 10 Biodiversity and Conservation Science, Department of Biodiversity, Conservation and Attractions, Kensington, WA Australia; 11 Centre for Australian National Biodiversity Research, Canberra, Australia; 12 National Seed Bank, Australian National Botanic Gardens, Department of Climate Change, Energy, the Environment and Water, Canberra, Australia; 13 School of Life Sciences, University of Technology Sydney, Broadway, Australia; 14 Australian PlantBank, Botanic Gardens of Sydney, Mount Annan, Australia; 15 School of Agriculture, Food and Ecosystem Sciences, University of Melbourne, Parkville, Australia; 16 Arthur Rylah Institute for Environmental Research, Victorian Department of Energy, Environment and Climate Action, East Melbourne, Australia; 17 School of Natural Sciences, Macquarie University, Macquarie Park, Australia; 18 Present Address: School of Natural Sciences, Macquarie University, Macquarie Park, Australia; 19 Present Address: Hawkesbury Institute for the Environment, Western Sydney University, Sydney, Australia

**Keywords:** Biodiversity, Ecophysiology, Fire ecology

## Abstract

Traits with intuitive names, a clear scope and explicit description are essential for all trait databases. The lack of unified, comprehensive, and machine-readable plant trait definitions limits the utility of trait databases, including reanalysis of data from a single database, or analyses that integrate data across multiple databases. Both can only occur if researchers are confident the trait concepts are consistent within and across sources. Here we describe the AusTraits Plant Dictionary (APD), a new data source of terms that extends the trait definitions included in a recent trait database, AusTraits. The development process of the APD included three steps: review and formalisation of the scope of each trait and the accompanying trait description; addition of trait metadata; and publication in both human and machine-readable forms. Trait definitions include keywords, references, and links to related trait concepts in other databases, enabling integration of AusTraits with other sources. The APD will both improve the usability of AusTraits and foster the integration of trait data across global and regional plant trait databases.

## Background & Summary

Large-scale analyses of trait data are now commonplace across many scientific disciplines, from vegetation modelling, to evolutionary dynamics and conservation planning^1,2^. At the broadest level, a trait is any morphological, physiological, chemical, or life history feature of an organism that can be documented^3^. Traits capture the enormous heterogeneity in form and function across individuals, populations, or taxonomic units. Variation in trait values reflects the ecological and evolutionary processes that give rise to functional diversity and, in turn, is thus used to define and describe units of biodiversity (e.g., species)^4^. For vascular plants, the increasing integration of big trait datasets into studies of plant ecology and evolution can be attributed to the rapid growth in databases that collate and/or harmonise collections of field-based observations for re-use^5^. Some plant trait databases are global^6–8^, while others have regional^9–12^ or taxonomic^13^ scopes. Some target specific organs or functions^14^, and others are more general, such as floras aimed at plant identification (e.g. http://www.worldfloraonline.org/). Combined with the growing interest in plant traits, the surge in available data is expanding our ability to answer a wide range of questions about the global flora^6,15^.

**Fig. 1:**
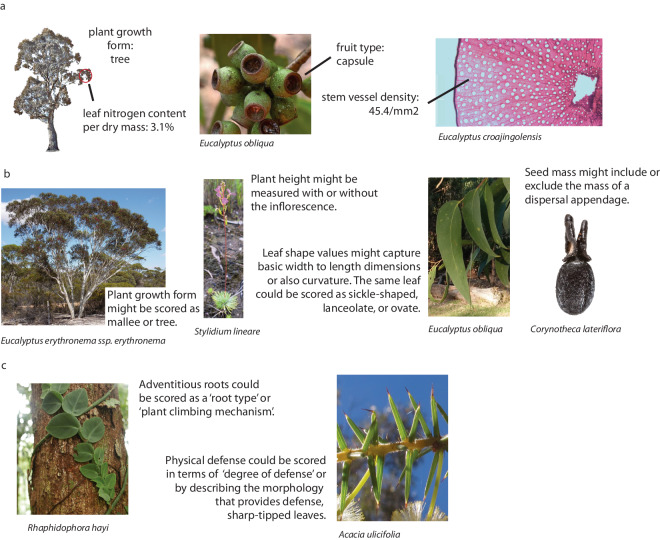
Explicit definitions and value descriptions are needed to reconcile inconsistencies in how researchers align plant phenotypic diversity with particular traits and trait values. (**a**) For some taxa, for some phenotype observations, all researchers are likely to assign the same observation to the same trait and trait value; (**b**) For other taxa, the same trait might not be consistently scored, especially without explicit definitions; (**c**) Some phenotypes will be aligned to different traits or different trait values by different researchers, especially if clear trait and trait value descriptions are not available. Photo credits: Russell Barrett (*Corynotheca lateriflora* seed); John Cull, iNaturalist (*Eucalyptus obliqua* leaves); Gillian Kowalick (*Eucalyptus croajingolensis* cross-section); Dean Nicolle, iNaturalist (*Eucalyptus erythronema* subsp. *erythronema*); Elizabeth Wenk (*Acacia ulicifolia*; *Rhaphidophora hayi*); Dylan Wishart, iNaturalist (*Eucalyptus obliqua* fruits); hughberry, iNaturalist (*Stylidium lineare*).

Usefully and accurately capturing the wonderful diversity of plant form and function to address ecological, biogeographic and evolutionary questions involves the non-trivial challenge of reconciling many and often conflicting definitions of plant traits. Garnier wrote of the “semantic bazaar” in trait ecology, referring to the diversity of possible meanings for a single trait name^16^. For instance, does plant height refer to vegetative height or the height of the highest inflorescence, the height of a typical adult or that of the tallest individual? Is leaf length the length of the leaf blade or does it include petiole length? Without definitions, data cannot be easily reused or merged across trait databases, as the trait names by themselves might not clearly indicate the “trait concept”^5^. Moreover, as each researcher sees the diversity of form and function in the natural world through a unique lens, the same physical feature on the same plant may be scored as being part of different trait concepts or given a different value of the same trait (Fig. 1).

Ideally, the relationships between different phenotypes and terms would be standardised, allowing researchers to easily reuse data in new contexts. Just as a taxon concept can be described as “a circumscribed set of organisms” (https://github.com/tdwg/tnc/issues/1), a trait concept delimits a collection of trait values pertaining to a distinct characteristic of a specific part of an organism (cell, tissue, organ, or whole organism). Trait names, like taxon names, are associated with each concept, attaching a reusable, interpretable label to each concept, but like taxon names require common terminology across research groups. Currently, research is hindered by the lack of explicit definitions outlining what trait concept a particular trait name refers to, what measurements a specific trait concept encompasses, and the difficulty of reconciling many plausible terms for a single phenotype.

As efforts towards data compilation and database integration have progressed, the need for explicit definitions is increasingly being recognised. Explicit, widely-adopted schemes have long existed for just a few traits (e.g. Raunkiaer’s life forms)^17^ and plant morphology books have long offered a rich vocabulary to describe plant parts^18,19^. Meanwhile, trait handbooks have emerged in the ecology and evolutionary biology literature as tools for standardising measurements and terminology^20–25^. Individual trait databases are also increasingly incorporating explicit trait definitions, enumerating allowable categorical trait values and linking their trait definitions to trait handbooks or published trait ontologies (Table 1)^7,10,16,26–28^. The Thesaurus Of Plant Characteristics (TOP; https://top-thesaurus.org/)^16^ was an initiative to define trait concepts for traits in the TRY plant trait database (Table 1)^6^. Still a work in progress, it was the first major effort to publish ecological plant trait definitions that included expected units, allowable categorical trait values and references. There also exist more formal vocabularies put forward by the Open Biological and Biomedical Ontology Foundry (OBO; https://obofoundry.org)^29^. One of the OBO Foundry ontologies, the Plant Trait Ontology (TO; https://bioportal.bioontology.org/ontologies/TO; Table 1)^30^, was the first extensive formal ontology of plant traits to be published, including definitions for hundreds of traits relevant to agricultural research organised into an intuitive hierarchy. EnvThes likewise offers a formally published ontology to support Long Term Ecological Research (LTER) data (https://vocabs.lter-europe.net/envthes/; Table 1)^31^ and is focused on ecological terms. The Crop Ontology is a collection of 39 individual ontologies, each for a specific crop, which include not only traits, but, for some traits, also measurement techniques, units, and the characteristic measured. While some Crop Ontologies are still modest in trait coverage, others, like the Woody Plants Ontology, include several hundred traits as well as terms related to the additional metadata fields. All these pioneering assemblies of trait definitions have advanced global integration of plant trait definitions, but these works remain incomplete relative to the breadth of trait concepts captured by large ecological trait databases.

**Table 1:** Information specified about trait concepts in a selection of trait thesauri, dictionaries, ontologies and databases.

	definitions	links to identical trait concepts	specifies units	specifies allowable ranges	specifies and defines allowable trait values	includes references	resolvable URIs for each trait
**Thesaurus, Dictionary or Ontology**							
AusTraits Plant Dictionary (APD)	YES	partially	YES	YES	YES	partially	YES
Crop Ontology (CO)	YES	partially	YES	NO	NO	partially	YES
Plant Trait Ontology (TO)	YES	rarely	NO	NO	partially	NO	YES
Thesaurus Of Plant Characteristics (TOP)	YES	NO	YES	NO	YES	partially	partially
Thesaurus for long term ecological research, monitoring and experiments (EnvThes)	partially	partially	NO	NO	NO	NO	YES
**Database**							
TRY	partially	NO	YES	NO	NO	NO	NO
GIFT	NO	NO	YES	NO	YES	NO	NO
BIEN	NO	NO	YES	NO	YES	NO	NO
LEDA	YES	NO	YES	YES	YES	YES	NO
BROT	YES	YES	YES	partially	YES	YES	NO

(citations: Crop Ontology^39^, Plant Trait Ontology^30^; TOP^16^; EnvThes^31^; TRY^6^; GIFT^7^; BIEN^8^; LEDA^26^; BROT 2.0^10^).

Meanwhile, the core components that should be encapsulated to fully define a trait concept have been established by emerging standards in bioinformatic platforms. They include the trait’s name (label), a concise but explicit description, standard units (for numeric traits) and allowable range (for numeric traits) or literal values (for categorical traits)^28,32–34^. Additional fields to enhance trait findability include keywords and a trait hierarchy. Interoperability and reusability are increased by including references and links to identical or similar traits in other trait databases. A further step toward making trait definitions FAIR (Findable, Accessible, Interoperable, Reproducible)^35^ is to add a resolvable identifier to each trait concept, explicitly linking each trait name to its published trait definition^28^, and to publish the output in both human and machine-readable forms.

**Fig. 2:**
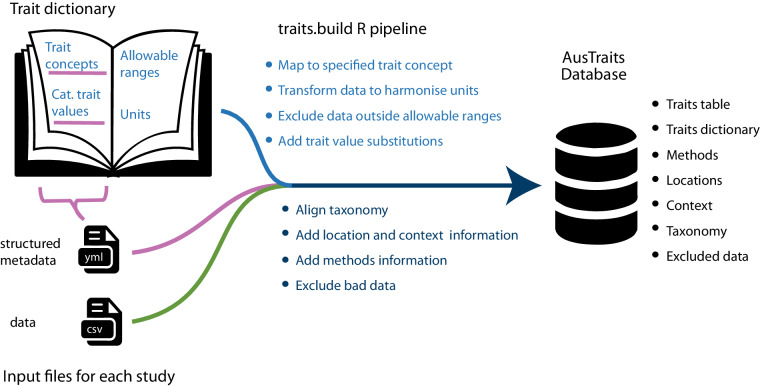
A trait dictionary is an essential component of the AusTraits workflow, specifying (i) trait concepts, (ii) standard units, (iii) allowable categorical trait values and (iv) allowable ranges for numeric traits. The structured metadata file that accompanies each dataset explicitly maps data columns to specific trait concepts from the dictionary and includes substitutions to align categorical trait values with those in the dictionary. The four elements of the trait definitions are then used by the traits.build R pipeline to integrate the data source into the AusTraits database.

Despite the multiple efforts from multiple groups, the ecological and systematics research communities currently lack comprehensive compilations of definitions that can be readily applied to new data. AusTraits^11^ is a large continental plant trait database that currently includes more than 1.7 million data records (v6.0.0) across nearly all of Australia’s 26,000 plant species. The 500 trait concepts required to characterise the AusTraits data encompass those related to plant morphology, fire ecology, life history, plant physiology, and nutrient content. We found that the existing trait dictionaries, thesauri, and ontologies did not document a sufficient breadth of traits, or omit information such as explicit trait descriptions, defined allowable categorical trait values, or allowable ranges (most comprehensive ones summarised in Table 1). As the project could not reuse an existing resource, the AusTraits team developed its own trait dictionary as the database grew (Fig. 2). The traits initially had informal definitions, which referenced existing vocabularies or references where possible. This process allowed the database to expand rapidly and efficiently, without being limited by availability of dictionaries, whilst still documenting trait definitions, preferred units, numeric range and allowable values for all traits in the database. While these definitions have allowed AusTraits users to interpret all data within the database and to manually link AusTraits data to those in other trait databases, it became apparent that a formal vocabulary would increase the utility of AusTraits and enhance researchers’ ability to reuse and integrate this and other data.

In this paper, we present the AusTraits Plant Dictionary (APD) – a comprehensive resource that documents traits of the Australian flora and enables linkages among datasets worldwide. The APD expands the original AusTraits trait definitions into a formal dictionary. We used a rigorous review process to devise and refine trait descriptions, included additional metadata with each definition, and released the trait dictionary in both human-friendly and machine-readable formats. Most of these definitions will also be useful in a global context, as they enhance what is available in existing resources. Our main aims were to create a vocabulary that:

1. Allowed all data within AusTraits to be effectively mapped onto semantically distinct trait concepts, enhancing the usability of AusTraits;
2. Linked trait concepts to other global trait resources, to facilitate analysis of AusTraits data in combination with data from other databases from around the world, and
3. Established trait definitions with resolvable identifiers, to allow the reuse of the APD definitions in other databases or vocabularies.

Building off prior efforts, the APD brings the plant trait ecology research community closer to a standardised approach for documenting the traits of the world’s flora. In addition to supporting AusTraits, our approach of reviewing and reconciling the often-conflicting trait concepts and descriptions and making them FAIR has created a resource that can be reused and built upon by other research initiatives in a global context. Research groups are already actively using this resource to facilitate the integration of trait data across trait databases^36^ and to add semantically clear definitions to newly tabulated trait values^37^. While a first version of the APD is now available, new versions will be released as we build upon the dictionary. We expect the approximately 500 traits and 800 categorical trait values included in the dictionary to expand over time, as the dictionary is used to map and interpret trait data for diverse databases and research projects, including those with global scope. For instance, the APD currently has only sparse coverage of root traits, completely lacks traits pertaining to tissue decomposition rates, and is missing some key traits in the hydraulics literature, as it has been built primarily in reference to the data currently in AusTraits. The current version of the APD occasionally lacks categorical trait values used globally, but which are not generally applied to the Australian flora (e.g. ‘xylopodium’ as a value for the trait ‘storage_organ’).

While this first version of the APD was developed by a team of ecologists and evolutionary biologists based in Australia, a future goal would be to incorporate additional categorical trait values (and additional traits), ensuring the trait definitions can be reused by any global dataset. Other future enhancements to broaden the global use of the APD may include trait labels in multiple languages (mapped in as synonyms) and trait descriptions in additional languages. Cross-cultural collaborations with Indigenous peoples who hold substantial knowledge of plant species and their characteristics would deepen the information content collated for each trait concept. A customised GitHub issue template allows researchers from across the plant trait research community to suggest additional traits to add to this initiative. A submission would include a proposed trait concept to add, a trait label and description, allowable ranges or values, and references. Once reviewed, these trait concepts could be included in future releases.

## Methods

There were three components to building a dictionary for these traits. First, we reviewed and revised each trait concept, minimising ambiguity in its scope and writing an explicit, yet concise trait description. Second, we added metadata fields to each trait definition. Third, we compiled all trait concepts into a single resource, output simultaneously in both human and machine-readable formats.

### Reviewing and revising trait concepts, an overview

#### Preliminary review

Through a preliminary review we divided all traits into three groups: (1) trait concepts that were clear and simple and could be reviewed by just the core AusTraits team; (2) trait concepts that required a brief review by experts; and (3) trait concepts where the trait’s scope or allowable values required significant discussion amongst experts; these were reviewed in a series of workshops (Table 2). For the 149 traits that were the content of an element, isotope, metabolite, or other biochemical compound in a specific plant organ, tissue or cell, the meaning and scope of the trait was usually unambiguous and universally agreed upon; few of these traits required a review outside the core AusTraits team. A review by just the core AusTraits team was also sufficient for 143 additional traits with very explicit, simple definitions, or that were trait concepts linked directly to a publication and accompanying dataset.

**Table 2:** Traits were divided into clusters requiring different styles of review (see Methods).

Review category	Example trait concepts	Count of traits in category
AusTraits team review	Leaf N per leaf dry mass, Bark water content per unit bark dry mass, Palisade cell length	292
Expert review	Leaf Jmax per unit leaf area, Sapwood specific conductivity (Ks), Pollen grain aperture shape	118
Workshop discussion	Plant growth form, Seed shape, Fire response, Storage organ, Leaf shape	112

#### Expert reviews

118 physiological and floral traits were reviewed by experts with extensive experience. These reviewers were able to efficiently identify unrealistic allowable value ranges, nonstandard units, incomplete trait descriptions and call attention to missing or inappropriate key references.

#### Workshop reviews

112 traits were allocated to a specialist workshop, generally because they contained long lists of synonymous or poorly defined categorical trait values or were traits measured differently by different groups of researchers. For traits that required an extensive review, we used a series of small (5–10 person) workshops that brought together researchers who would ideally apply an identical trait concept to diverse research situations. The workshops included researchers at government agencies, universities or herbaria; researchers who were functional ecologists, taxonomists or systematists; and researchers with expertise in diverse plant communities. Three workshops were conducted covering traits within the realms of ‘Seed and dispersal traits’ (October 2021), ‘Leaf and whole plant vegetative traits’ (August 2022) and ‘Fire response and regeneration traits’ (May 2023); each was comprised of 4 or 5 two-hour workshop sessions. Moderated by AusTraits team members, each session was dedicated to clarifying the trait concepts, refining the trait descriptions, identifying key references and carefully compiling a list of allowable trait values that was succinct and distinct. The trait workshops identified trait concepts that were too vague, trait concepts that lacked semantic clarity and curated categorical trait values.

### Completing trait definitions

The core goal of all reviews was to delineate trait concepts and lists of trait values to which all data submitted to AusTraits could be unambiguously mapped. The outcomes of the workshops, expert reviews and internal reviews were used to write a trait description and propagate additional metadata for each trait concept.

#### Trait descriptions and comments

A clear, explicit and comprehensive trait description was drafted for each trait. Whenever possible, trait descriptions were closely aligned to those in trait handbooks, reference books, research papers describing key methods and existing trait ontologies. Following the example set by formal ontologies (e.g. the OBO Foundry ontologies PATO, PO and TO) and the TOP Thesaurus, a second formal description was drafted for each trait where all technical terms were linked to classes (words; concepts) in published ontologies. This removed ambiguity in what was meant by a term and required that all definitions were written with reference to a narrow list of words. Although this formal method generated a unique definition for each trait, the less formal, non-annotated trait descriptions were considered essential to convey the trait concepts to users, as the encoded descriptions are often awkward to read and interpret. In addition, a comments field provided a location to document notes, including referencing similar traits, best practice measurement methods, important context variables and possible sources of error within the amalgamated data. For instance, the definition for the trait “leaf area” could include a comment indicating that although only leaf area data are meant to be mapped to this trait concept, many authors will merge leaflet and leaf area data under the title “leaf area” and therefore trait databases, such that AusTraits, will contain a mix of leaf and leaflet area data under the “leaf area” trait. Meanwhile, for photosynthetic rate traits the comments field could indicate that it is best practise to document leaf temperature as a context property.

In addition to the trait description and comments, descriptive metadata fields were added to each trait concept (Table 3)^38^. The metadata fields include those required for data processing (e.g. allowable ranges and allowable trait values), those that increase trait concept findability (e.g. keywords), those that properly document the source and scope of the trait (e.g. references, reviewers) and those that increase trait concept interoperability across datasets (e.g. matches to traits in other databases or ontologies).

**Table 3:** Metadata provided for each trait concept, including a description of each metadata field and the published annotation property onto which this information is mapped.

APD Field	Description	Type	Annotation property*	Example for fruit length
Identifiers and Labels				
Label	A concise English label for the trait	Text string	SKOS:label	Fruit length
AusTraits database label	A label for the trait where words are connected by underscores	Text string	SKOS:altLabel	fruit_length
Trait ID	A numeric identifier	Text string	dcterms:identifier	trait_0012512
Descriptions				
Description	A pair of descriptions, ranging from 1–3 sentences that clearly indicates the trait’s scope. One description is written in plain English and for the second, technical terms are linked to published ontologies whenever possible.	Text string	dcterms:description	Linear dimension from the base to the apex of a fresh fruit, even if this is not the longest dimension.; A fruit morphology trait [TO:0002629] which is the length [PATO:0000122] of a fresh [EnvThes:21976] fruit [PO:0009001] from the fruit proximal end [PO:0008002] (base) to the fruit distal end [PO:0008001] (apex).
Comments	Additional notes about the scope of the trait or acceptable methods.	Text string	RDFS:comment	(none)
Metadata required for processing				
Type	Type of trait, specifying if traits are categorical, numeric, or ordinal	Entity IRI**	ETS:valueType	continuous variable
Units	The preferred units for the trait, conforming to the Unified Code for Units of Measure (UCUM). There are often two entries for units, one that is a string and the second which links to a units of measurement axiom.	Entity IRI and/or Text string	ETS:expectedUnit	mm
Allowed values min & Allowed values max	A lower and upper boundary for accepted numerical values	Number	ETS:minAllowedValue & ETS:maxAllowedValue	0.01 - 2000
Allowed values levels	Allowable terms (trait values) for categorical traits	Text string	ETS:factorLevels***	
Metadata to increase trait concept findability				
Measured structure	Indication of what organ(s), tissue(s), or other plant structure is being measured for a given trait	Entity IRI	iadopt:hasContextObject	Fruit; reproductive shoot system
Measured characteristic	Keywords pertaining to what categorical or numeric property is measured, such as whether the measurement is a length, volume, duration of time, or shape	Entity IRI	oboe-core:MeasuredCharacteristic	Length; size
Keyword	Additional descriptors beyond the trait category, tissue entity and measured characteristic which facilitate information retrieval	Entity IRI and/or text string	SIO:000147 (keyword)	reproduction
Metadata to increase trait concept documentation				
References	Key sources for trait concept, scope and definition	Entity IRI	dcterms:references	Pérez-Harguindeguy 2013; Kew Seed Information Database 2022
Scope of trait concept	The scope of the trait, specifying taxonomic or morphological groupings to which the trait concept applies	Text string	SKOS:scopeNote	
Date created	The date when the trait metadata was first created	Date	dcterms:created (date created)	14/07/2021
Date modified	The date when the trait metadata was last revised	Date	dcterms:modified (date modified)	02/05/2024
Date reviewed	The date when the trait definition and scope of the trait were reviewed.	Date	dcterms:reviewed (date reviewed)	30/11/2021
Previous trait labels	Trait labels previously used for this trait concept	Text string	SKOS:changeNote	
Reviewer	People who have reviewed the trait concept, identified by ORCID number	Entity IRI	datacite/4.4:isReviewedBy	Elizabeth Wenk, Hervé Sauquet, Russell Barrett, Carl Gosper, Lydia Guja, Gregory J. Jordan, Mark Ooi, Karen D. Sommerville, Lily Dun
Metadata to increase trait concept interoperability				
Exact match	Identical trait concepts in other formally published ontologies	Entity IRI	SKOS:exactMatch	obo:TO_0002626
Close match	Similar trait concepts in other formally published ontologies	Entity IRI	SKOS:closeMatch	
Related match	Related trait concepts in other formally published ontologies	Entity IRI	SKOS:relatedMatch	
Example	Identical, similar or related trait concepts in trait databases or informally published ontologies	Text string	SKOS:example	Fruit length [TOP92] (https://top-thesaurus.org/index)
				Fruit length [TRY:918] (https://www.try-db.org/de/de.php)
				fruit_length [GIFT:3.13] (https://gift.uni-goettingen.de)
				maximum fruit length; minimum fruit length [BIEN] (https://bien.nceas.ucsb.edu/bien/biendata)

*The annotation properties indicate the source ontology for each field, with the abbreviations linked to the source vocabularies as follows: SKOS = http://www.w3.org/2004/02/skos/core#; dcterms = http://purl.org/dc/terms/; RDFS = http://www.w3.org/2000/01/rdf-schema#; ETS = http://terminologies.gfbio.org/terms/ETS/; uom = https://w3id.org/uom/; iadopt = https://w3id.org/iadopt/ont/; oboe-core = http://ecoinformatics.org/oboe/oboe.1.2/oboe-core.owl#; SIO = http://semanticscience.org/resource/; datacite = http://purl.org/datacite/v4.4/

**‘Entity IRI’ indicates that the information within this field is a term that has its own URL (e.g. references, reviewers ORCIDs) or Internationalized Resource Identifier (IRI; for terms in a published vocabulary/ontology).

***Allowable categorical trait values (allowable levels) are mapped in APD as SKOS: narrower (for each trait).

#### Metadata required for processing

The {traits.build} R package that compiles AusTraits requires five pieces of information for each trait concept: the label (i.e., a trait name), the trait type (numeric versus categorical), the allowable range of values for numeric traits, the standardised units for numeric traits, and the allowable trait values for categorical traits (Fig. 2; Table 3).

#### Metadata to increase trait concept findability

Metadata should also include descriptors that aid in the discovery of the resource, here the individual trait concepts. In addition to offering a trait hierarchy, APD includes three fields to increase trait findability: the plant structure being measured (e.g., stem, leaf, root, whole plant, flower), the characteristic being measured (e.g., mass, shape, force) and additional keywords (Table 3).

#### Metadata to increase trait concept documentation

Each trait in the APD includes metadata to record the date trait concepts were described and revised; the people involved in reviewing each trait concept and trait definition; its applicability (i.e., scope; the trait might only be scorable for specific taxonomic groups or in plants that have leaves); and past labels (names) used for the identical trait concept. In addition, references were linked to traits whenever possible; these included trait handbooks describing the trait, manuscripts introducing or championing the trait and review papers noting the best traits to measure to document a particular plant function. A field links the standardised units to published vocabularies, described below.

#### Metadata to increase trait concept interoperability

A cluster of metadata elements promote the interoperability of this resource with other databases by documenting trait concepts in other trait databases or ontologies that are identical, similar, or related to a specific trait concept in the APD (Table 3). Trait concepts from TRY^6^, TOP^16^, GIFT^7^, LEDA^26^, BIEN^8^, BROT 2.0^10^, the Palm Traits Database^13^, the Plant Trait Ontology^30^, the Woody Plant Ontology^39^ and EnvThes^31^ were cross-mapped to trait concepts in the APD.

#### Mapping metadata fields to concepts in published vocabularies

In a standard tabular format, the metadata fields are the column headers, each specifying a different piece of information documented about the trait. In a formal ontology, each metadata field must be matched to an appropriate annotation property. These are published, formally defined terms for ‘label’, ‘description’, etc. (Table 3). By preference, metadata fields in the APD are linked to concepts defined by the often-used Simple Knowledge Organization System (SKOS; https://www.w3.org/TR/skos-primer/)^40^), Resource Description Framework (RDF; https://www.w3.org/TR/rdf11-primer/)^41^, or Dublin Core Metadata Initiative (dcterms; https://www.dublincore.org/specifications/dublin-core/dcmi-terms)^42^ vocabularies. Properties defined by the Ecological Trait-data Standard (ETS)^34^ were also reused, as this schema is establishing itself as a well-designed ecological trait database structure. Units were aligned to the Unified Code for Units of Measure (UCUM; https://ucum.org/ucum)^43^ standard with specific machine-readable representations of each unit downloaded from the Units of Measurement (UOM) portal (https://units-of-measurement.org/). The UCUM standard follows clear, simple rules, but also has a flexible syntax for documenting notes that are recorded as part of the ‘unit’ for specific traits, yet are not formally units, in curly brackets^44^. For instance, {count}/mm2 or umol{CO2}/m2/s, where the actual units are 1/mm2 and umol/m2/s, respectively. An added advantage is that the UOM representations include links to identical units in a collection of other published ontologies. Properties not present in any of these ontologies were mapped to ones in the Semantic Science Information Ontology (SIO)^45^, the Extensible Observation Ontology (OBOE)^46^ and Datacite^47^ (Table 3).

Within each APD trait concept, some trait metadata fields were simply text strings, while other metadata values were themselves published concepts with a Uniform Resource Identifier (URI) (an inclusive term, that encompasses both URLs and Internationalized Resource Identifiers [IRIs]). For instance, matches to exact, close, and related trait concepts in formally published ontologies have URIs, references mostly have a DOI (digital object identifier), reviewers were identified by their ORCID (Open Researcher and Contributor Identifier) and keywords were all identified by their URIs from various published ontologies. Meanwhile, trait description, trait labels, and matches to traits in ecological databases that lack formally published definitions were mapped as text strings (literals).

### Trait hierarchy

For ease of grouping trait concepts, we established a trait hierarchy into which the traits could be slotted. At the highest level, all traits within the APD could be divided into four categories: biochemical traits, morphological traits, physiological traits and life history traits (Table S1). Three of these were exact matches to classes defined by the Plant Trait Ontology^30^ (plant biochemical trait (http://purl.obolibrary.org/obo/TO_0000277); plant morphology trait (http://purl.obolibrary.org/obo/TO_0000017); and biological process trait, physiological process trait (http://purl.obolibrary.org/obo/TO_0000283)), while life history trait was defined within APD. Additional hierarchical levels were established, again using a combination of terms from the Plant Trait Ontology and ones defined within APD (Table S1). The trait hierarchy was mapped into the formal ontology as ever narrower concepts, cascading down from the top concept, a Plant Trait (http://purl.obolibrary.org/obo/TO_0000000)^30^.

### Adding resolvable identifiers

Each term defined within the APD requires a unique and stable URI, such that other databases and ontologies can reference individual APD trait concepts, just as some of the trait concepts within the APD are now mapped to previously published trait concepts. This includes not just the trait concepts, but also the allowable categorical trait values, the trait groupings within the trait hierarchy, and the selection of terms within a glossary. We chose to register the APD namespace with the URI redirection service w3id.org (https://w3id.org/) to ensure the permanency of the URIs even if the project’s GitHub repository were to be moved. The trait concepts, trait groupings for the hierarchy, and allowable categorical trait values are within one schema, https://w3id.org/APD/traits/ while the glossary terms are in a second schema, https://w3id.org/APD/glossary. The APD profile with the w3id.org redirect service references the current versioned APD release (https://github.com/perma-id/w3id.org/blob/master/APD/.htaccess).

### Compiled input tables

Eight separate tables were established to compile the metadata associated with trait definitions, including four associated with APD trait concepts and four documenting information about classes from published vocabularies that are linked to the APD (URIs, labels, and other metadata for references, reviewers, keywords, and units). A further three tables were required to document 1) the base URIs for the published vocabularies from which classes are reused by the APD; 2) the URIs of the published properties linked to each column header; and 3) the base URIs for the APD vocabulary itself. The resulting tables are as follows.

**APD_traits_input.csv** is the core data table into which trait labels, trait descriptions and all associated metadata for each trait concept were mapped (Table S2). As indicated in Table 3, some columns are textual strings, others are numeric and some refer to pre-existing entities (concepts, classes). The pre-existing entities are documented in an additional four data tables, APD_references.csv, APD_reviewers.csv, APD_units.csv and published_classes.csv. As required for mapping into a formal vocabulary, each cell includes a single unit of information or semicolon-delimited lists of information and, where applicable, indicates an identifier for a term in another vocabulary, rather than its label.

**APD_categorical_values_input.csv** contains the allowable trait values for each categorical trait, including descriptions of each term and indicating the trait concept to which the term is linked (Table S3).

A challenge in the compilation of APD was that formal vocabularies (ontologies) allow only a single instance of each word to be used, with a single definition. While each trait name is unique, the same term (word) can be used as a categorical trait value for multiple traits with subtly different meanings and possibly different meanings to a pre-existing ontology. Generalising the definitions to be applicable to all instances of its use would mean that its definition would be far broader than implied as a specific trait value for a single categorical trait. The solution for APD was for official trait values to be the merging of the trait label and the term, while the label for the term could be a simple word that might be reused. For instance, *hairy* is an allowed value for five separate traits. While “hairy” remains the label for all instances of its use, for the trait *Juvenile phase leaf hairiness* the formal trait value becomes *leaf_hairs_juvenile_leaves_hairy*.

**APD_trait_hierarchy.csv** indicates the hierarchical structure into which the trait concepts are mapped (Table S4).

**APD_glossary.csv** includes a collection of terms used repeatedly within APD trait concept descriptions or as keywords, but which lacked an appropriate published definition (Table S5).

**APD_references.csv** links each reference indicated in APD_traits.csv to its DOI (or alternative identifier), also providing a title and complete reference (as a string) (Table S6).

**APD_reviewers.csv** links each reviewer indicated in APD_traits.csv to their ORCID number (Table S7).

**APD_units.csv** links the standardised units indicated in APD_traits.csv to their respective URLs in the Units of Measurement Ontology (Table S8). The data table includes a description of the unit, links to its SI and UCUM representation and indicates other ontologies with definitions for this unit.

**published_classes.csv** documents terms from published ontologies used as keywords, measured characteristics, measured structures, or to describe the trait type. The label, description, IRI, scheme URI and scheme prefix are provided for each term (Table S9).

**APD_namespace_declaration.csv** indicates the URI for each vocabulary prefix referenced in APD_traits.csv and serves as the namespace declaration when compiling the RDF representation (Table S10).

**APD_annotation_properties.csv** indicates the source, label and description for each of the annotation properties (Table 3) used to capture metadata for the trait concepts (Table S11).

**APD_resource.csv** includes the annotation properties that describe the core APD resources, APD/traits and APD/glossary (Table S12).

### Review of trait concepts and allowable trait values

In total, the APD includes 522 traits, including 112 categorical traits and 410 numeric traits (Table S13). These vary from well-known traits like leaf area to bespoke traits like leaf pendulousness that are measured only for specific research questions. The internal reviews, expert reviews by individuals, and reviews through the trait workshops all worked toward clarifying trait concepts and developing clear trait descriptions and appropriate lists of allowable categorical trait values. The key categories of issues identified were as follows.

#### Trait concept, label and description too vague

The vocabulary workshops uncovered several instances where trait names were ambiguous and may have led to the misinterpretation of data. For instance, the trait ‘leaf angle’ was defined as the angle between the stem and the leaf blade, but it was identified that the data in AusTraits referred to the leaf blade’s angle relative to the solar zenith. As a result, there are now two traits in the APD with more explicit labels and definitions, leaf axil angle and leaf inclination angle. Another example of a semantically unclear trait label was the trait capturing the hairiness of juvenile leaves. It was unclear if these were the leaves on a juvenile plant or the juvenile (regrowth) leaves on an adult plant following disturbance. Again, it was necessary to adopt two separate traits whose scopes were more explicit. In addition, by linking the terms in the trait description to ontologies, it was possible to clearly distinguish between a leaf on a ‘juvenile plant’ (http://purl.obolibrary.org/obo/PATO_0001190)^48^ versus a ‘juvenile leaf’ (http://purl.obolibrary.org/obo/PO_0006339)^30^ on an adult plant.

#### Trait concept too broad

**Fig. 3:**
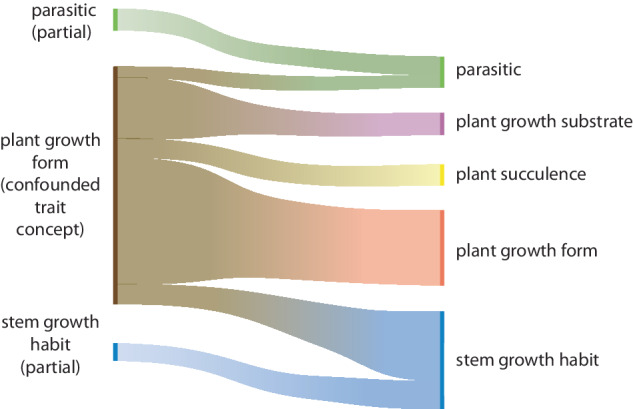
There were initially 68 trait values mapped to the semantically messy trait concept ‘plant growth form’ and incomplete trait concepts ‘parasitic’ and ‘stem growth habit’ (left). For the refined trait concepts (right), these were able to be condensed to 53, despite adding more detailed trait values to parasitic, plant succulence and stem growth habit traits. For the APD, the retained trait values were mapped across five traits: plant growth form, plant succulence, plant growth substrate, parasitic and stem growth habit. The mixing of semantic concepts within ‘plant growth form’ had previously resulted in hybrid terms which could now be eliminated, such as “shrub_aquatic”.

There were several traits that were identified as being too broad and including two (or more) semantically distinct concepts; these traits were split into multiple traits with a narrower, explicitly defined scope. For instance, fruit type included both true, botanical fruit types and terms that simply indicated whether a dispersal unit was dry or fleshy. The data initially merged together under fruit type were split into a trait that captured true botanical fruit types, such as achenes and drupes^49^, and then two traits that indicated specific functions of the fruit, independent of its formal classification, i.e., fruit fleshiness and fruit dehiscence. Plant growth form included terms that pertained not only to the actual entire plant form, but also values indicating whether a species was terrestrial, aquatic, epiphytic or parasitic. The initial scope of data mapped to plant growth form was divided into a simpler plant growth form which was focused on the plant’s perennating 3-dimensional shape, with ancillary information mapped to plant growth substrate, plant succulence and, in part, a revised stem growth habit and parasitic traits (Fig. 3). Trait concepts that are too broad are a global problem and other trait databases have also recently taken the approach of splitting plant growth form into more tractable traits with a clearly defined ‘entity’ and scope^7,50^. For the APD, this allowed a considerable reduction in repetitive trait values, such as remapping ‘aquatic_herbs’; ‘aquatic_shrubs’ and ‘aquatic_trees’ to ‘herbs’, ‘shrubs’ and ‘trees’ under plant growth form and as ‘aquatic’ under plant growth substrate.

#### Curating categorical trait values

Certain categorical traits were identified as those most requiring standardisation of trait values and were selected to review during the workshops. These included seed shape, fruit type, dispersal syndrome, leaf shape, leaf type, resprouting capacity, and plant growth form. These were traits for which there were data in many datasets, but which lacked universally agreed upon allowable trait values. For instance, despite attempts to condense terms and align meanings, AusTraits had 50–80 trait values for leaf shape; many were clearly synonymous terms or terms not actually related to the shape of the leaf blade. There were two core reasons for these long lists of terms: (1) traits that integrated data from both the ecology and the systematics communities, with different researchers favouring different sets of terms; and (2) the lack of available vocabulary to describe particular trait phenotypes.

Plant morphologists and taxonomists are equipped with botanical glossaries^18,19,51,52^, offering a detailed vocabulary to describe all nuances of a plant’s morphology. In contrast, while ecologists use these morphological terms when appropriate, ecology datasets also include terms that capture specific functional roles, often using a merging of formal and informal terms. By curating categorical trait values, two core revisions were made. The first was to condense the extensive list of terms in botanical glossaries. Although many researchers in these fields take advantage of this rich descriptive vocabulary, they were amenable to reducing the list of terms allowed as values for a given trait, realising that the fine-grained distinctions were unlikely to have functional significance, but also that many terms were so similar they were unlikely to be used consistently, even by the experts. This concurs with recent research that suggests that all people, even expert botanists, were more likely to correctly identify a plant’s character when there were fewer options to choose from^53^. Synonymous terms were listed within the description of each trait value, clarifying the scope of each trait value retained and facilitating searches for terms that were omitted. For example, for seed surface texture, the final list included 11 trait values, but an additional 28 terms were mapped as synonyms (Table 4). For some traits, appropriate lists of terms were discovered through literature searches or emerged through workshop discussions. For instance, the many leaf shape values could easily be mapped to the terms in a resource established by the Systematics Association Committee 60 years ago^54^, which was not known to most but familiar to one workshop participant.

**Table 4:** Explicitly listing synonyms as part of trait value definitions ensured alternative terminology can be consistently mapped to the term used in the APD, as illustrated here for seed surface texture, https://w3id.org/APD/traits/trait_0012620.

Trait Value	Synonyms
bumpy	colliculate, verrucate, papillate, tuberculate, undulate
grooved	
netted	reticulate, honey-combed
papery	chartaceous
pitted	foveolate, foveate, dimpled, lacunose, punctate
ribbed	carinate, costate, fluted, lineate, lineolate, ridged, scalariform, striate, strigose
rough	scabrous
scaly	scurfy, squarrose
smooth	glabrous
spiny	echinulate
wrinkled	rugose, rugulose, bullate

Some challenges emerged when selecting a list of allowable words to describe the ecological or functional trait values where no succinct, unified list of terms exists. The difficulty is exemplified by plant growth form, where even successive versions of the same trait handbook presented barely overlapping lists of allowable growth form terms^20,21^, despite this being one of the most recorded traits worldwide. These resources and many others share the use of ‘tree’, ‘shrub’ and ‘herb’, but beyond these terms resources diverge in their list of allowable plant growth form values. Our list uses terms from both of these references as well as many others^7,16,50^, compiling a list onto which all existing AusTraits data could be mapped. Our goal was to balance having enough terms to capture morphological and functional diversity, while allowing for comparative analyses across groupings. For plant growth form, as for other traits, this list included terms of ecological or descriptive significance that might be used only for specific taxa or ecological situations, yet were required for trait measurements in those circumstances. For the Australian flora, terms like ‘mallee’ and ‘hummock’ were deemed essential to describe distinct plant growth forms, although these terms are absent or rarely used globally. Meanwhile the term ‘treelet’, in common use in tropical floras outside Australia, was omitted from this release of the APD, but may be included in the future to encourage global reuse of the trait concept. In the final list there was a clear scope and description attached to each trait value.

### Compiling output data products

The eight input tables were propagated with all required metadata for the trait concepts, categorical trait values, and to describe the APD namespace. Terms from 39 published vocabularies were used as metadata for some property within the APD (Table S14). R scripts were developed to merge the input files into the final data outputs (v4.3.1)^55^. Within R, we used packages from tidyverse (dplyr, tidyr, readr, stringr, purrr)^56^ for data manipulation and knitr^57^, kableExtra^58^, and gt^59^ to generate web outputs.

### Building the APD into a single data table

An R script allowed all metadata for the trait concepts and categorical trait values to be compiled into a compact pair of tables. These two tables allow the trait concepts and their metadata to be easily reused by researchers wishing to programmatically integrate a collection of trait definitions into trait databases, trait concept descriptions for a manuscript, or meta-analyses integrating information from multiple sources. In particular, encoded terms from ontologies are accompanied by their English labels, multiple columns documenting instances of the same metadata (i.e. reviewers) were collapsed into a single column, and more intuitive names were given to metadata columns (Table 5). The categorical trait values are output as a second table (Table 6).

**Table 5:** Columns in the data table APD_traits.csv.

Column	Description
Entity	IRI for trait within APD schema
trait	Trait concept (alternate trait label)
label	Trait name (label for trait within APD schema)
description	Description of trait.
comments	Additional comments about the trait, including possible sources of error, related traits, or best-practise methodologies.
trait_type	String indicating whether this is a categorical or numeric trait.
allowed_values_min	For numeric traits, the minimum allowable value.
allowed_values_max	For numeric traits, the maximum allowable value.
units	For numeric traits, the standard units for this trait within APD.
constraints	The scope of the trait, indicating taxonomic groups for which the trait is used or if the trait only applies to taxa with specific morphologies.
trait_groupings	Hierarchical categories into which the trait is mapped.
structure_measured	The plant structure (a tissue, organ, or the whole plant) that is measured by this trait.
characteristic_measured	The characteristic that is measured, such as whether the trait records ‘mass’, ‘shape’, ‘length’, etc.
keywords	Keywords linked to this trait concept.
references	References linked to this trait concept.
reviewers	People who have reviewed this trait concept.
created	Date the trait was first created.
modified	Date the trait was most recently modified.
reviewed	Date the trait definition and scope of the trait was reviewed.
exact_match	Matches to identical traits in formally published ontologies.
close_match	Matches to similar traits in formally published ontologies.
related_match	Matches to related traits in formally published ontologies.
example	Matches to identical, similar, and related traits in trait databases and informally published ontologies, thesauri or dictionaries.
description_encoded	Description of trait, with keywords linked to terms from published vocabularies/ontologies.
deprecated_trait_name	Previous labels used for this trait concept
identifier	Numeric identifier within the APD scheme.
inScheme	Indication that this term is within the ADP schema

**Table 6:** Columns in the data table APD_categorical_values.csv.

Column	Description
allowed_values_levels	The label for an allowable trait value for a categorical APD trait concept.
trait	A specific categorical APD trait concept.
categorical_trait_description	A description of the allowed categorical trait value.
categorical_trait_synonyms	Synonyms for the allowed categorical trait value.
categorical_trait_identifier	The identifier within the APD schema for the allowed categorical trait value. (This field merges the categorical trait value label and the trait name to ensure each categorical trait value within the APD is a unique concept.)

### Building the APD into a machine-readable resource

The formal output for the trait concepts and their associated metadata needed to be in a machine-readable format. This was built using an R script that first merged the eight separate data tables into a single table formatted as RDF Triples, the core unit of the Resource Description Framework (RDF) data model. With the triples format, all information content is collapsed into a single long-format document with three columns, the subject, the predicate, and the object. The subject is always the URI for a concept. For the APD these are the resolvable identifiers within the w3id.org namespace generated for each APD trait concept, categorical trait value, and trait hierarchy category. Also included were previously published concepts that were referenced as trait metadata within the APD, such as ORCIDs for reviewers, DOI’s for references, or URIs for concepts reused from published vocabularies. The predicate indicates a property of the object that is being described. The predicates in AusTraits are the annotation properties in Table 3 and additional terms specified under ‘Column’ in Tables S2–S12. Each predicate is also a URI. The object is the value for the specific predicate for the specific object and can be either a URI or a text string.

Terms sourced from published ontologies or other sources were mapped into the APD as their own entities to ensure their labels were included within the APD, as otherwise they could only be identified within the APD by a label-less URI. As each value of a property of an object is in a new row in triples format, there may be more than 30 rows of data for a single trait concept, and, in total, there are more than 22,000 rows of unique object-predicate-value combinations within the APD.

The R package rdflib^60^ was used to serialise the table of triples into RDF objects, output in Turtle (APD.ttl), N-Triple (APD.nt), N-Quad (APD.nq) and JSON Linked Data (APD.json) formats.

**Fig. 4:**
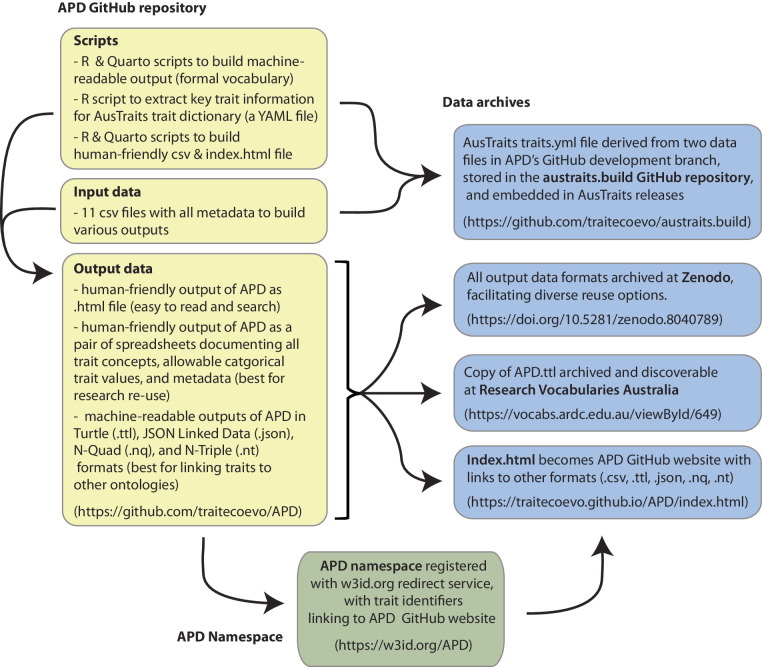
The APD scripts, inputs and output data are stored on the project’s GitHub repository. Versioned releases are archived at Zenodo and the Australian Data Research Common’s Research Vocabulary Australia (RVA) portal. APD has been registered as a namespace within w3id.org, with term URIs redirecting back to an html landing page within the GitHub repository. The APD inputs are also used to generate the traits.yml file required to build the AusTraits trait database.

The RDF serialisations were complemented by two derivatives, created from the N-Triple output using a combination of R and Quarto scripts (Fig. 4). The first is a HTML landing page for human interaction with the machine-readable formats (https://traitecoevo.github.io/APD/index.html) to which all searches for individual concept URIs are automatically redirected. And second is the YAML (.yml) file required by the AusTraits workflow to compile the database. It includes only the trait labels, trait description, type, allowable range, allowable trait values and required units and is located within the austraits.build GitHub repository (https://github.com/traitecoevo/austraits.build). The YAML format offers a flexible data serialisation format to capture diverse metadata in a single file, as it has a nested format which allows different numbers of levels beneath each header. This permits both easy data input and human interpretation.

## Data Records

The APD Zenodo version 2.0.0 release^61^ includes the seven data resources derived from the input tables (10.5281/zenodo.8040789). These input tables and the scripts required to build the data records (outputs) are archived on the APD GitHub repository (https://github.com/traitecoevo/APD) and contain the information used to build the data records (outputs) (Fig. 4). The data are available under a CC-BY 4.0 license, allowing reuse with attribution. These data records include identical content compiled into formats suited to different use cases:

**APD_traits.csv** is a derived tabular output that condenses the information in the APD input tables into a single table, in a format well-suited to researchers. All metadata for each trait is contained within a single row and all information pertaining to a specific annotation property is collapsed into a single cell, with both an English label and the identifier linking the label to a published vocabulary (Table 5).

**APD_categorical_values.csv** is a derived tabular output that includes the allowable values for all categorical traits, to be used in conjunction with APD_traits.csv. Each value has a description and, where appropriate, an additional column documents synonymous terms (Table 6).

**APD_triples.csv** is a tabular output format where the Objects (values) of all Predicates (annotation properties) of all Subjects (concepts; classes) are presented in triples format. This is a long output structure whereby each row is a single Subject-Predicate-Object combination. All RDF representations and the index.html file are derived from this file using an R script available on the projects GitHub repository (https://github.com/traitecoevo/APD) (Table S15).

**index.html** offers a human-readable compiled version of the information contained in APD_triples.csv. It is the same information hosted at https://traitecoevo.github.io/APD/.

**APD.json,** **APD.nq,** **APD.nt**, and **APD.ttl** are four different RDF representations of the information contained in APD_triples.csv. Among these, APD.ttl (a ‘turtle’ file) is considered to be the most readable. These representations allow the individual APD trait concepts to be integrated into ontology servers and read and re-used by researchers building other ontologies (Table S16). In particular, APD.ttl was published online through the Australian Research Data Common’s (ARDC) Research Vocabularies Australia (https://ardc.edu.au/services/research-vocabularies-australia/).

The version controlled machine-readable Turtle representation is also published through Research Vocabularies Australia, part of the national research infrastructure operated by the Australian Research Data Commons (ARDC) (https://vocabs.ardc.edu.au/viewById/649)^62^. The APD GitHub repository (https://github.com/traitecoevo/APD) has both versioned releases and ongoing development versions. The APD namespace (w3id.org/APD) and trait concept URIs (e.g. https://w3id.org/APD/traits/trait_0000014) redirect to the versioned releases on the APD GitHub repository.

A document on the project’s GitHub website includes example code to search and extract data from the APD (https://traitecoevo.github.io/APD/using_the_APD.html).

## Technical Validation

The APD.ttl file (Turtle serialisation) was run through a skos validator to confirm that all relationships were consistent, all URIs were unique, and that all concepts have labels. The file APD_triples.csv was used to recompile the HTML landing page. The APD_traits_input.csv and APD_categorical_values_input.csv files were used to recompile the inputs of the AusTraits workflow. Deriving the HTML output from the Turtle serialization and confirming AusTraits continued to build properly from the automatically regenerated YAML file, confirmed the files were complete and the process was accurate.

### Supplementary information

Supplementary Tables

## Data Availability

The code to compile the data into the selected output formats is available in the APD GitHub repository (https://github.com/traitecoevo/APD).
