# Supplementary material for: The AusTraits plant dictionary
Source: Sci Data. 2024 May 25;11:537. doi: 10.1038/s41597-024-03368-z (PMC11127939; doi:10.1038/s41597-024-03368-z)
Supplement: Supplementary file 1 — Supplementary Tables [file 41597_2024_3368_MOESM1_ESM.pdf]

Supplementary Tables

Contents

Supplementary Tables 1

Table S1. Number of traits in each of the hierarchical trait groupings. 2

Table S2. Columns in the input table APD_traits_input.csv 4

Table S3. Columns in the input table APD_categorical_values_input.csv........ 6

Table S4. Columns in the input table APD_trait_hierarchy.csv........ 6

Table S5. Columns in the input table APD_glossary.csv........ 7

Table S6. Columns in the input table APD_references.csv........ 7

Table S7. Columns in the input table APD_reviewers.csv 7

Table S8. Columns in the input table APD_units.csv 8

Table S9. Columns in the input table published_classes.csv........ 8

Table S10. Columns in the input table APD_namespace_declaration.csv 8

Table S11. Columns in the input table APD_annotation_properties.csv 9

Table S12. Columns in the input table APD_resource.csv........ 9

Table S13. Traits within the APD. 10

Table S14. Published vocabularies from which classes (terms) were sourced as metadata for a property within the APD. 29

Table S15. Columns in the data table APD_triples.csv 31

Table S16. Output for the trait `life history` from APD.ttl........ 32

Table S1. Number of traits in each of the hierarchical trait groupings.

trait grouping	number of trait concepts within group*
**biochemical trait**	
**mineral and ion content trait**	
leaf mineral and ion content trait	
live leaf mineral and ion content trait	33
senesced leaf mineral and ion content trait	16
stem mineral and ion content trait	17
wood mineral and ion content trait	7
senesced wood mineral and ion content trait	6
bark mineral and ion content trait	9
root mineral and ion content trait	3
reproductive shoot system mineral and ion content trait	13
cell or tissue mineral and ion content trait	14
**metabolite content trait**	
carbohydrate content trait	13
lipid content trait	1
phenolic compound content trait	5
pigment content trait	9
protein content trait	4
**stable isotope ratio determination**	14
**plant morphology trait**	
**whole plant morphology trait**	5
plant embryo morphology trait	8
**plant structure morphology trait**	6
portion of plant tissue morphology trait	11
plant cell morphology trait	6
**leaf morphology trait**	30
leaf size trait	11
leaf mass trait	16
leaf shape trait	10
leaf position trait	6
leaf stomatal complex morphology trait	6
leaf optical properties trait	4
**stem morphology trait**	34
stem mass trait	6
**bark morphology trait**	9
**root system morphology trait**	14
**reproductive shoot system morphology trait**	3

floral organ morphology trait	7
perianth morphology trait	12
androecium morphology trait	14
gynoecium morphology trait	8
fruit morphology trait	13
seed morphology trait	16
**vascular tissue morphology trait**	5
leaf vein morphology trait	4
xylem vessel morphology trait	10
**plant structure strength trait**	10
**biological process trait, physiological process trait**	
photosynthetic trait	2
gas exchange trait	13
photosynthetic rate trait	7
respiration rate trait	4
transpiration rate trait	6
carbon dioxide concentration trait	6
photosystem performance trait	11
water transport trait, hydraulic trait	53
nutrient recycling trait	2
**life history trait**	
whole plant phenotype trait	16
plant phenological trait	16
interspecific interactions trait	8
genetic structure trait	2
reproductive structure life history trait	15
fire response trait	32
chemical stress sensitivity trait	3
environmental tolerance trait	16

* The total number of traits in this table is greater than the total number of traits in APD, since some traits appear in multiple categories.

Table S2. Columns in the input table APD_traits_input.csv

Column(s)	Description
identifier	IRI for trait within APD schema
trait	Alternate label for trait within APD schema
label	Label for trait within APD schema
description_encoded	Description of trait, with key words linked to terms from published vocabularies/ontologies
description	Description of trait
comments	Additional comments about the trait, including possible sources of error, related traits, or best-practise methodologies
inScheme	Indication that this term is within the ADP schema
type	Indicating whether this is a categorical or numeric trait, by linking to the appropriate term within the STATO ontology
min	For numeric traits, the minimum allowable value
max	For numeric traits, the maximum allowable value
units	For numeric traits, the standard units for this trait within APD
units_uom	For numeric traits, the units of measurement syntax for the standard units
category	Hierarchical categories into which the trait is mapped
created	Date the trait was first created
modified	Date the trait was most recently modified
reviewed	Date the trait was reviewed
deprecated_trait_name	Previous labels used for this trait concept
constraints	The scope of the trait, indicating taxonomic groups for which the trait is used or if the trait only applies to taxa with specific morphologies
structure	The plant structure (a tissue, organ, or the whole plant) that is measured by this trait
measured_characteristic	The characteristic that is measured, such as whether the trait records \`mass\`, \`shape\`, \`length\`, etc.
reviewers	People who have reviewed this trait concept
references	References linked to this trait concept
keywords	Keywords linked to this trait concept; the keywords are generally terms in published vocabularies
exact_match, close_match, related_match	Formally published vocabularies/ontologies with traits that are identical, similar, or related to this trait concept.
TOP_exact, TOP_close, TOP_related	Traits within the TOP Trait Thesaurus that are identical, similar, or related to this trait concept. These are mapped as examples of the trait concept.
TRY_exact, TRY_close, TRY_related	Traits within the TRY Plant Trait Database that are identical, similar, or related to this trait concept. These are mapped as examples of the trait concept.

LEDA_exact, LEDA_close, LEDA_related	Traits within the LEDA Database that are identical, similar, or related to this trait concept. These are mapped as examples of the trait concept.
GIFT_exact, GIFT_close, GIFT_related	Traits within the GIFT Database that are identical, similar, or related to this trait concept. These are mapped as examples of the trait concept.
BIEN_exact, BIEN_close, BIEN_related	Traits within the BIEN Database that are identical, similar, or related to this trait concept. These are mapped as examples of the trait concept.
BROT_exact, BROT_close, BROT_related	Traits within the BROT Database that are identical, similar, or related to this trait concept. These are mapped as examples of the trait concept.
PalmTraits_exact, PalmTraits_close	Traits within the PalmTraits Database that are identical or similar to this trait concept. These are mapped as examples of the trait concept.

Table S3. Columns in the input table APD_categorical_values_input.csv

Column	Description	Annotation Property*
identifier	Identifier for a specific categorical trait value within APD	dcterms:identifier
label	Label for the categorical trait value	skos:label
description	Description of the categorical trait value	dcterms:description
trait_name	Trait name to which the categorical trait value refers	skos:broader

* See Table 3 footnotes for the full schema URL's associated with each annotation property prefix.

Table S4. Columns in the input table APD_trait_hierarchy.csv

Column	Description	Annotation Property*
Entity	URI for a trait category (hierarchical level) within APD	
label	Label for a trait category (hierarchical level) within APD	skos:label
description	Description of a trait category (hierarchical level) within APD	dcterms:description
Parent	Superclass (higher hierarchical level) for a trait category within APD	skos:broader
exactMatch	Link to identical concept in a published ontology	skos:exactMatch
tier_1	Highest hierarchical level into which the category fits	*(Not used as an annotation property)*
tier_2	Second highest hierarchical level into which the category fits	*(Not used as an annotation property)*
tier_3	Third highest hierarchical level into which the category fits (if applicable)	*(Not used as an annotation property)*
tier_4	Fourth highest hierarchical level into which the category fits (if applicable)	*(Not used as an annotation property)*
hierarchy	Written string indicating the full hierarchy of the specific category	*(Not used as an annotation property)*

* See Table 3 footnotes for the full schema URL's associated with each annotation property prefix.

Table S5. Columns in the input table APD_glossary.csv

Column	Description	Annotation Property*
identifier	Identifier for a specific glossary term within APD/glossary	dcterms:identifier
label	Label for the glossary term	skos:label
description	Description of the glossary term	dcterms:description

* See Table 3 footnotes for the full schema URL's associated with each annotation property prefix.

Table S6. Columns in the input table APD_references.csv

Column	Description	Annotation Property*
Entity	URL for reference, if available	
label	Author-year label for reference	skos:label
citation	Full reference citation	dcterms:bibliographicCitation
identifier	Identifier for reference, a DOI when available, or otherwise an ISBN (for books) or URL (for websites)	dcterms:identifier
title	The title of the reference	dcterms:title

* See Table 3 footnotes for the full schema URL's associated with each annotation property prefix.

Table S7. Columns in the input table APD_reviewers.csv

Column	Description	Annotation Property*
Entity	URL for the reviewer's ORCID profile	
label	Reviewer's full name	skos:label
ORCID	Reviewer's ORCID number	obo:IAO_0000708 (ORCID identifier)

* See Table 3 footnotes for the full schema URL's associated with each annotation property prefix.

Table S8. Columns in the input table APD_units.csv

Column	Description	Annotation Property*
Entity	Units of Measurement IRI for the specific units of measurement	
label	Label for these units of measurement	skos:label
altLabel	Alternative written label for these units of measurement	skos:altLabel
description	Verbal description of these units of measurement	dcterms:description
SI_code	The International System of Units code for these units of measurement	uom:SI_code
UCUM_code	The Unified Code for Units of Measure code for these units of measurement	uom:UCUM_code
exactMatch	Up to 6 columns indicating exact matches for this unit of measurement in other ontologies	skos:exactMatch

* See Table 3 footnotes for the full schema URL's associated with each annotation property prefix.

Table S9. Columns in the input table published_classes.csv

Column	Description	Annotation Property*
Entity	URI for a specific term (class) in a published ontology	
label	Label for the term	skos:label
description	Verbal description of the term	(Not used)
identifier	Identifier for the term within a specific vocabulary	dcterms:identifier
inScheme	URI for the vocabulary in which the term is published	skos:inScheme
prefix	Prefix for the specific vocabulary	*(Not used as an annotation property)*
vocabulary	Name of the specific vocabulary	*(Not used as an annotation property)*

* See Table 3 footnotes for the full schema URL's associated with each annotation property prefix.

Table S10. Columns in the input table APD_namespace_declaration.csv

Column	Description	Annotation Property*
prefix	Prefix used for a specific vocabulary within APD machine-readable representations	
Scheme	URI for each vocabulary used in the APD	skos:inScheme

* See Table 3 footnotes for the full schema URL's associated with each annotation property prefix.

Table S11. Columns in the input table APD_annotation_properties.csv

Column	Description	Annotation Property*
Entity	URI for annotation properties used by the APD	
label	Label for annotation properties used by the APD, from its own vocabulary	skos:label
description	Description of annotation properties used by the APD, from its own vocabulary	dcterms:description
issued	Date a term was issued within its vocabulary	dcterms:issued
comment	Additional comments about annotation properties used by the APD, from its own vocabulary	rdfs:comment
isDefinedBy	URI for the vocabulary in which the term is published	rdfs:isDefinedBy
inScheme	URI for the vocabulary in which the term is published	skos:inScheme

* See Table 3 footnotes for the full schema URL's associated with each annotation property prefix.

Table S12. Columns in the input table APD_resource.csv

Column	Description
Subject	Entity URI for the APD schema
Predicate	Annotation properties for the APD schema
Object	Value of a particular annotation property for the APD schema

Table S13. Traits within the APD.

The URI for each trait is w3id.org/APD/traits/XXX, where XXX is the APD identifier. Full details for each trait are provided at these links.

Label	alternate label (AusTraits `trait_name`)	APD identifier
**Biochemical Traits**		
Leaf aluminium (Al) content per unit leaf dry mass	leaf_Al_per_dry_mass	trait_0000012
Leaf boron (B) content per unit leaf dry mass	leaf_B_per_dry_mass	trait_0000014
Leaf carbon (C) content per unit leaf dry mass	leaf_C_per_dry_mass	trait_0000016
Leaf calcium (Ca) content per unit leaf dry mass	leaf_Ca_per_dry_mass	trait_0000018
Leaf chlorine (Cl) content per unit leaf dry mass	leaf_Cl_per_dry_mass	trait_0000020
Leaf chromium (Cr) content per unit leaf dry mass	leaf_Cr_per_dry_mass	trait_0000022
Leaf cobalt (Co) content per unit leaf dry mass	leaf_Co_per_dry_mass	trait_0000024
Leaf copper (Cu) content per unit leaf dry mass	leaf_Cu_per_dry_mass	trait_0000026
Leaf iron (Fe) content per unit leaf dry mass	leaf_Fe_per_dry_mass	trait_0000028
Leaf potassium (K) content per unit leaf area	leaf_K_per_area	trait_0000029
Leaf potassium (K) content per unit leaf dry mass	leaf_K_per_dry_mass	trait_0000030
Leaf magnesium (Mg) content per unit leaf dry mass	leaf_Mg_per_dry_mass	trait_0000032
Leaf manganese (Mn) content per unit leaf dry mass	leaf_Mn_per_dry_mass	trait_0000034
Leaf molybdenum (Mo) content per unit leaf dry mass	leaf_Mo_per_dry_mass	trait_0000036
Leaf nitrogen (N) content per unit leaf area	leaf_N_per_area	trait_0000037
Leaf nitrogen (N) content per unit leaf dry mass	leaf_N_per_dry_mass	trait_0000038
Leaf sodium (Na) content per unit leaf dry mass	leaf_Na_per_dry_mass	trait_0000040
Leaf nickel (Ni) content per unit leaf dry mass	leaf_Ni_per_dry_mass	trait_0000042

Leaf phosphorus (P) content per unit leaf area	leaf_P_per_area	trait_0000043
Leaf phosphorus (P) content per unit leaf dry mass	leaf_P_per_dry_mass	trait_0000044
Leaf sulphur (S) content per unit leaf dry mass	leaf_S_per_dry_mass	trait_0000046
Leaf selenium (Se) content per unit leaf dry mass	leaf_Se_per_dry_mass	trait_0000048
Leaf silicon (Si) content per unit leaf dry mass	leaf_Si_per_dry_mass	trait_0000050
Leaf zinc (Zn) content per unit leaf dry mass	leaf_Zn_per_dry_mass	trait_0000052
Leaf carbon to nitrogen ratio (C/N)	leaf_CN_ratio	trait_0000090
Leaf nitrogen to phosphorus ratio (N/P) per unit leaf dry mass	leaf_NP_ratio	trait_0000091
Senesced leaf aluminium (Al) content per unit leaf dry mass	leaf_senesced_Al_per_dry_mass	trait_0000112
Senesced leaf boron (B) content per unit leaf dry mass	leaf_senesced_B_per_dry_mass	trait_0000114
Senesced leaf carbon (C) content per unit leaf dry mass	leaf_senesced_C_per_dry_mass	trait_0000116
Senesced leaf calcium (Ca) content per unit leaf dry mass	leaf_senesced_Ca_per_dry_mass	trait_0000118
Senesced leaf copper (Cu) content per unit leaf dry mass	leaf_senesced_Cu_per_dry_mass	trait_0000126
Senesced leaf iron (Fe) content per unit leaf dry mass	leaf_senesced_Fe_per_dry_mass	trait_0000128
Senesced leaf potassium (K) content per unit leaf dry mass	leaf_senesced_K_per_dry_mass	trait_0000130
Senesced leaf magnesium (Mg) content per unit leaf dry mass	leaf_senesced_Mg_per_dry_mass	trait_0000132
Senesced leaf manganese (Mn) content per unit leaf dry mass	leaf_senesced_Mn_per_dry_mass	trait_0000134
Senesced leaf molybdenum (Mo) content per unit leaf dry mass	leaf_senesced_Mo_per_dry_mass	trait_0000136
Senesced leaf nitrogen (N) content per unit leaf dry mass	leaf_senesced_N_per_dry_mass	trait_0000138
Senesced leaf sodium (Na) content per unit leaf dry mass	leaf_senesced_Na_per_dry_mass	trait_0000140
Senesced leaf nickel (Ni) content per unit leaf dry mass	leaf_senesced_Ni_per_dry_mass	trait_0000142

Senesced leaf phosphorus (P) content per unit leaf dry mass	leaf_senesced_P_per_dry_mass	trait_0000144
Senesced leaf sulphur (S) content per unit leaf dry mass	leaf_senesced_S_per_dry_mass	trait_0000146
Senesced leaf zinc (Zn) content per unit leaf dry mass	leaf_senesced_Zn_per_dry_mass	trait_0000152
Leaf nitrogen resorption	leaf_N_resorption	trait_0022012
Leaf phosphorus resorption	leaf_P_resorption	trait_0022013
Stem carbon (C) content per unit stem dry mass	stem_C_per_dry_mass	trait_0000216
Stem nitrogen (N) content per unit stem dry mass	stem_N_per_dry_mass	trait_0000238
Wood carbon (C) content per unit wood dry mass	wood_C_per_dry_mass	trait_0000416
Wood calcium (Ca) content per unit wood dry mass	wood_Ca_per_dry_mass	trait_0000418
Wood potassium (K) content per unit wood dry mass	wood_K_per_dry_mass	trait_0000430
Wood magnesium (Mg) content per unit wood dry mass	wood_Mg_per_dry_mass	trait_0000432
Wood nitrogen (N) content per unit wood dry mass	wood_N_per_dry_mass	trait_0000438
Wood sodium (Na) content per unit wood dry mass	wood_Na_per_dry_mass	trait_0000440
Wood phosphorus (P) content per unit wood dry mass	wood_P_per_dry_mass	trait_0000444
Dead wood calcium (Ca) content per unit dead wood dry mass	wood_dead_Ca_per_dry_mass	trait_0000518
Dead wood potassium (K) content per unit dead wood dry mass	wood_dead_K_per_dry_mass	trait_0000530
Dead wood magnesium (Mg) content per unit dead wood dry mass	wood_dead_Mg_per_dry_mass	trait_0000532
Dead wood nitrogen (N) content per unit dead wood dry mass	wood_dead_N_per_dry_mass	trait_0000538
Dead wood sodium (Na) content per unit dead wood dry mass	wood_dead_Na_per_dry_mass	trait_0000540
Dead wood phosphorus (P) content per unit dead wood dry mass	wood_dead_P_per_dry_mass	trait_0000544
Bark aluminium (Al) content per unit bark dry mass	bark_Al_per_dry_mass	trait_0000612

Bark boron (B) content per unit bark dry mass	bark_B_per_dry_mass	trait_0000614
Bark carbon (C) content per unit bark dry mass	bark_C_per_dry_mass	trait_0000616
Bark calcium (Ca) content per unit bark dry mass	bark_Ca_per_dry_mass	trait_0000618
Bark copper (Cu) content per unit bark dry mass	bark_Cu_per_dry_mass	trait_0000626
Bark iron (Fe) content per unit bark dry mass	bark_Fe_per_dry_mass	trait_0000628
Bark potassium (K) content per unit bark dry mass	bark_K_per_dry_mass	trait_0000630
Bark magnesium (Mg) content per unit bark dry mass	bark_Mg_per_dry_mass	trait_0000632
Bark manganese (Mn) content per unit bark dry mass	bark_Mn_per_dry_mass	trait_0000634
Bark nitrogen (N) content per unit bark dry mass	bark_N_per_dry_mass	trait_0000638
Bark sodium (Na) content per unit bark dry mass	bark_Na_per_dry_mass	trait_0000640
Bark phosphorus (P) content per unit bark dry mass	bark_P_per_dry_mass	trait_0000644
Bark sulphur (S) content per unit bark dry mass	bark_S_per_dry_mass	trait_0000646
Bark zinc (Zn) content per unit bark dry mass	bark_Zn_per_dry_mass	trait_0000652
Root carbon (C) content per unit root dry mass	root_C_per_dry_mass	trait_0000816
Root nitrogen (N) content per unit root dry mass	root_N_per_dry_mass	trait_0000838
Root phosphorus (P) content per unit root dry mass	root_P_per_dry_mass	trait_0000844
Flower nitrogen (N) content per unit flower dry mass	flower_N_per_dry_mass	trait_0001038
Fruit calcium (Ca) content per unit fruit dry mass	fruit_Ca_per_dry_mass	trait_0001118
Fruit potassium (K) content per unit fruit dry mass	fruit_K_per_dry_mass	trait_0001130
Fruit magnesium (Mg) content per unit fruit dry mass	fruit_Mg_per_dry_mass	trait_0001132

Fruit nitrogen (N) content per unit fruit dry mass	fruit_N_per_dry_mass	trait_0001138
Fruit phosphorus (P) content per unit fruit dry mass	fruit_P_per_dry_mass	trait_0001144
Fruit sulphur (S) content per unit fruit dry mass	fruit_S_per_dry_mass	trait_0001146
Seed calcium (Ca) content per unit seed dry mass	seed_Ca_per_seed_dry_mass	trait_0001218
Seed potassium (K) content per unit seed dry mass	seed_K_per_seed_dry_mass	trait_0001230
Seed magnesium (Mg) content per unit seed dry mass	seed_Mg_per_seed_dry_mass	trait_0001232
Seed nitrogen (N) content per unit seed dry mass	seed_N_per_seed_dry_mass	trait_0001238
Seed phosphorus (P) content per unit seed dry mass	seed_P_per_seed_dry_mass	trait_0001244
Seed sulphur (S) content per unit seed dry mass	seed_S_per_seed_dry_mass	trait_0001246
Leaf cell wall nitrogen (N) per unit cell wall dry mass	leaf_cell_wall_N_per_cell_wall_dry_mass	trait_0001511
Leaf cell wall nitrogen (N) per unit leaf N content	leaf_cell_wall_N_per_leaf_N	trait_0001512
Leaf rubisco nitrogen (N) content per unit leaf N content	leaf_rubisco_N_per_total_leaf_N	trait_0001513
Leaf thylakoid protein nitrogen (N) content per unit leaf N content	leaf_thylakoid_N_per_total_leaf_N	trait_0001514
Leaf epidermis calcium (Ca) content per unit leaf fresh mass	leaf_epidermis_Ca_per_fresh_mass	trait_0001611
Leaf hypodermis calcium (Ca) content per unit leaf fresh mass	leaf_hypodermis_Ca_per_fresh_mass	trait_0001612
Leaf internal parenchyma cell calcium (Ca) content per unit leaf fresh mass	leaf_internal_parenchyma_Ca_per_fresh_mass	trait_0001613
Leaf palisade mesophyll cell calcium (Ca) content per unit leaf fresh mass	leaf_palisade_mesophyll_Ca_per_fresh_mass	trait_0001614
Leaf sclerenchyma cell calcium (Ca) content per unit leaf fresh mass	leaf_sclerenchyma_Ca_per_fresh_mass	trait_0001615
Leaf spongy mesophyll cell calcium (Ca) content per unit leaf fresh mass	leaf_spongy_mesophyll_Ca_per_fresh_mass	trait_0001616
Leaf epidermis phosphorus (P) content per unit leaf fresh mass	leaf_epidermis_P_per_fresh_mass	trait_0001661

Leaf hypodermis phosphorus (P) content per unit leaf fresh mass	leaf_hypodermis_P_per_fresh_mass	trait_0001662
Leaf internal parenchyma cell phosphorus (P) content per unit leaf fresh mass	leaf_internal_parenchyma_P_per_fresh_mass	trait_0001663
Leaf palisade mesophyll cell phosphorus (P) content per unit leaf fresh mass	leaf_palisade_mesophyll_P_per_fresh_mass	trait_0001664
Leaf sclerenchyma cell phosphorus (P) content per unit leaf fresh mass	leaf_sclerenchyma_P_per_fresh_mass	trait_0001665
Leaf spongy mesophyll cell phosphorus (P) content per unit leaf fresh mass	leaf_spongy_mesophyll_P_per_fresh_mass	trait_0001666
Leaf total non-structural carbohydrate content per unit leaf area	leaf_total_non-structural_carbohydrates_per_area	trait_0002021
Leaf total non-structural carbohydrate content per unit leaf dry mass	leaf_total_non-structural_carbohydrates_per_mass	trait_0002022
Leaf cellulose content per unit leaf dry mass	leaf_cellulose_per_dry_mass	trait_0002024
Leaf starch content per unit leaf area	leaf_starch_per_area	trait_0002025
Leaf soluble starch content per unit leaf area	leaf_soluble_starch_per_area	trait_0002027
Leaf soluble starch content per unit leaf dry mass	leaf_soluble_starch_per_mass	trait_0002028
Leaf soluble sugar content per unit leaf area	leaf_soluble_sugars_per_area	trait_0002031
Leaf soluble sugar content per unit leaf dry mass	leaf_soluble_sugars_per_mass	trait_0002032
Leaf soluble protein content per unit leaf area	leaf_soluble_protein_per_area	trait_0002035
Leaf insoluble protein content per unit leaf area	leaf_insoluble_protein_per_area	trait_0002037
Leaf lignin content per unit leaf dry mass	leaf_lignin_per_dry_mass	trait_0002050
Total leaf phenolic content per unit leaf dry mass	leaf_phenol_per_dry_mass	trait_0002052
Leaf tannin content per unit leaf dry mass	leaf_tannin_per_dry_mass	trait_0002054
Leaf carotenoid content per unit leaf area	leaf_carotenoid_per_area	trait_0002055
Leaf carotenoid content per unit leaf dry mass	leaf_carotenoid_per_dry_mass	trait_0002056
Leaf total chlorophyll content (chlorophyll A + B) per unit leaf area	leaf_chlorophyll_per_area	trait_0002081
Leaf total chlorophyll content (chlorophyll A + B) per unit leaf dry mass	leaf_chlorophyll_per_dry_mass	trait_0002082

Leaf chlorophyll A content per unit leaf area	leaf_chlorophyll_A_per_area	trait_0002083
Leaf chlorophyll A content per unit leaf dry mass	leaf_chlorophyll_A_per_dry_mass	trait_0002084
Leaf chlorophyll B content per unit leaf area	leaf_chlorophyll_B_per_area	trait_0002085
Leaf chlorophyll B content per unit leaf dry mass	leaf_chlorophyll_B_per_dry_mass	trait_0002086
Ratio of leaf chlorophyll A content to leaf chlorophyll B content	leaf_chlorophyll_A_B_ratio	trait_0002087
Leaf rubisco content per unit leaf dry mass	leaf_rubisco_per_leaf_dry_mass	trait_0002090
Stem soluble starch content per unit stem dry mass	stem_soluble_starch_per_mass	trait_0002127
Stem soluble sugar content per unit stem dry mass	stem_soluble_sugars_per_mass	trait_0002131
Bark cellulose content per unit bark dry mass	bark_cellulose_per_dry_mass	trait_0002224
Bark lignin content per unit bark dry mass	bark_lignin_per_dry_mass	trait_0002250
Bark tannin content per unit bark dry mass	bark_tannin_per_dry_mass	trait_0002255
Root soluble starch content per unit root dry mass	root_soluble_starch_per_mass	trait_0002327
Root soluble sugar content per unit root dry mass	root_soluble_sugars_per_mass	trait_0002331
Seed protein content per unit seed dry mass	seed_protein_per_seed_dry_mass	trait_0002534
Seed oil content per unit seed dry mass	seed_oil_per_seed_dry_mass	trait_0002544
Leaf ash content per unit leaf dry mass	leaf_ash_per_dry_mass	trait_0002822
Bark ash content per unit bark dry mass	bark_ash_per_dry_mass	trait_0002824
Bark stable carbon isotope composition (delta13C)	bark_delta13C	trait_0003011
Leaf stable carbon isotope composition (delta13C)	leaf_delta13C	trait_0003012
Stem stable carbon isotope composition (delta13C)	stem_delta13C	trait_0003013
Root stable carbon isotope composition (delta13C)	root_delta13C	trait_0003014
Wood stable carbon isotope composition (delta13C)	wood_delta13C	trait_0003015
Bark stable nitrogen isotope composition (delta15N)	bark_delta15N	trait_0003031

Leaf stable nitrogen isotope composition (delta15N)	leaf_delta15N	trait_0003032
Stem stable nitrogen isotope composition (delta15N)	stem_delta15N	trait_0003033
Root stable nitrogen isotope composition (delta15N)	root_delta15N	trait_0003034
Wood stable nitrogen isotope composition (delta15N)	wood_delta15N	trait_0003035
Leaf xylem stable nitrogen isotope composition (delta15N)	leaf_xylem_delta15N	trait_0003052
Root xylem stable nitrogen isotope composition (delta15N)	root_xylem_delta15N	trait_0003053
Leaf stable oxygen isotope composition (delta18O)	leaf_delta18O	trait_0003072
Stem water stable oxygen isotope composition (delta18O)	stem_water_delta18O	trait_0003092
**Plant Morphology Trait**		
Plant canopy width	plant_width	trait_0010021
Plant canopy breadth	plant_breadth	trait_0010022
Plant vegetative height	plant_height	trait_0010023
Stem diameter at breast height	plant_diameter_breast_height	trait_0010024
Stem count	stem_count	trait_0010025
Plant spinescence	plant_spinescence	trait_0010070
Embryo colour	embryo_colour	trait_0010110
Cotyledon function	cotyledon_function	trait_0010111
Cotyledon position at germination	cotyledon_position	trait_0010112
Cotyledon hairiness	cotyledon_hairs	trait_0010113
Hypocotyl hairiness	seedling_hypocotyl_hairs	trait_0010114
Seedling first true leaf type	seedling_first_node_leaf_type	trait_0010160
Seedling first node leaf count	seedling_first_node_leaf_count	trait_0010161
Seedling germination location	seedling_germination_location	trait_0010162
Leaf area	leaf_area	trait_0011211
Leaflet area	leaflet_area	trait_0011212
Leaf length	leaf_length	trait_0011213
Leaf width	leaf_width	trait_0011214
Leaf thickness	leaf_thickness	trait_0011215
Leaf dry mass	leaf_dry_mass	trait_0011216
Leaflet dry mass	leaflet_dry_mass	trait_0011217
Leaf fresh mass	leaf_fresh_mass	trait_0011218
Petiole length	petiole_length	trait_0011219

Petiole width	petiole_width	trait_0011220
Leaf mass per area	leaf_mass_per_area	trait_0011230
Leaf lamina mass per area	leaf_lamina_mass_per_area	trait_0011231
Leaf tissue density	leaf_density	trait_0011232
Leaf area ratio (LAR)	leaf_area_ratio	trait_0011260
Leaf mass fraction	leaf_mass_fraction	trait_0011261
Leaf dry matter content (LDMC)	leaf_dry_matter_content	trait_0011262
Leaf fresh mass per leaf area	leaf_fresh_mass_per_area	trait_0011263
Leaf water content per unit leaf area (leaf succulence)	leaf_water_content_per_area	trait_0011264
Leaf water content per unit leaf dry mass	leaf_water_content_per_dry_mass	trait_0011265
Leaf water content per unit leaf fresh mass	leaf_water_content_per_fresh_mass	trait_0011266
Leaf water content per unit saturated leaf mass	leaf_water_content_per_saturated_mass	trait_0011267
Leaf cell wall fraction	leaf_cell_wall_fraction	trait_0011268
Leaf type	leaf_type	trait_0011310
Leaf shape	leaf_shape	trait_0011311
Leaf base shape	leaf_base_shape	trait_0011312
Leaf margin	leaf_margin	trait_0011313
Leaf margin posture	leaf_margin_posture	trait_0011314
Leaf lobation	leaf_lobation	trait_0011315
Leaf compoundness	leaf_compoundness	trait_0011316
Leaf divisions	leaf_lamina_division	trait_0011317
Leaf lamina posture (leaf 3-dimensionality)	leaf_posture_numeric	trait_0011318
Leaf lamina posture (leaf 3-dimensional shape)	leaf_lamina_posture	trait_0011319
Leaf glaucousness	leaf_glaucousness	trait_0011360
Mature leaf hairiness	leaf_hairs_adult_leaves	trait_0011361
Juvenile phase leaf hairiness	leaf_hairs_juvenile_leaves	trait_0011362
Immature leaf hairiness	leaf_hairs_immature_leaves	trait_0011363
Leaf phyllotaxis	leaf_phyllotaxis	trait_0011410
Leaf arrangement	leaf_arrangement	trait_0011411
Leaf axil angle	leaf_axil_angle	trait_0011412
Leaf inclination angle	leaf_inclination_angle	trait_0011413
Leaf pendulousness	leaf_pendulousness	trait_0011414
Cuticle thickness on the lower leaf surface	leaf_cuticle_thickness_abaxial	trait_0011510
Cuticle thickness on the upper leaf surface	leaf_cuticle_thickness_adaxial	trait_0011511
Leaf epidermis thickness	leaf_epidermis_thickness	trait_0011512
Lower leaf side epidermis thickness	leaf_epidermis_thickness_abaxial	trait_0011513
Upper leaf side epidermis thickness	leaf_epidermis_thickness_adaxial	trait_0011514

Average leaf epidermal cell density	leaf_epidermal_cell_density_both_sides	trait_0011515
Lower leaf side epidermal cell density	leaf_epidermal_cell_density_abaxial	trait_0011516
Upper leaf side epidermal cell density	leaf_epidermal_cell_density_adaxial	trait_0011517
Lower leaf side hypodermis thickness	leaf_hypodermis_thickness_abaxial	trait_0011518
Upper leaf side hypodermis thickness	leaf_hypodermis_thickness_adaxial	trait_0011519
Lower palisade mesophyll thickness	leaf_palisade_tissue_thickness_abaxial	trait_0011520
Upper palisade mesophyll thickness	leaf_palisade_tissue_thickness_adaxial	trait_0011521
Palisade cell length	leaf_palisade_cell_length	trait_0011522
Palisade cell width	leaf_palisade_cell_width	trait_0011523
Number of layers of palisade cells	leaf_palisade_layer_number	trait_0011524
Spongy mesophyll cell thickness	leaf_spongy_mesophyll_thickness	trait_0011525
Cell cross-sectional area	cell_cross-sectional_area	trait_0011526
Stomatal density on the lower leaf surface	leaf_stomatal_density_abaxial	trait_0011610
Stomatal density on the upper leaf surface	leaf_stomatal_density_adaxial	trait_0011611
Stomatal density averaged across both leaf surfaces	leaf_stomatal_density_average	trait_0011612
Stomatal distribution	leaf_stomatal_distribution	trait_0011613
Stomatal hairiness	leaf_stomatal_hairs	trait_0011614
Guard cell length	leaf_guard_cell_length	trait_0011615
Leaf visible light transmission	leaf_transmission	trait_0011710
Leaf visible light absorption	leaf_absorption	trait_0011711
Leaf visible light reflection	leaf_reflectance	trait_0011712
Leaf infra-red light reflection	leaf_reflectance_near_infrared	trait_0011713
Stem cross-sectional area	stem_cross_sectional_area	trait_0011811
Wood cross-sectional area	sapwood_cross_sectional_area	trait_0011812
Terminal twig cross-sectional area	branch_terminal_twig_cross_sectional_area	trait_0011813
Terminal twig length	branch_terminal_twig_length	trait_0011814
Wood density	wood_density	trait_0011815
Herbaceous stem density	stem_density	trait_0011816
Huber value	huber_value	trait_0011911
Leaf dry mass to stem dry mass ratio	leaf_mass_to_stem_mass_ratio	trait_0011912
Stem dry mass to vegetative shoot dry mass ratio (support fraction)	stem_mass_to_shoot_mass_ratio	trait_0011913
Side branch dry mass to whole plant dry mass ratio	branch_mass_fraction	trait_0011914
Stem dry matter content (SDMC)	stem_dry_matter_content	trait_0011915
Stem water content per unit saturated stem mass	stem_water_content_per_saturated_mass	trait_0011916
Stem mass fraction	stem_mass_fraction	trait_0011917
Bark morphology, Eucalyptus	bark_morphology_eucalyptus	trait_0012010

Bark thickness	bark_thickness	trait_0012011
Scaled bark thickness	bark_thickness_index	trait_0012012
Bark density	bark_density	trait_0012013
Bark dry mass per unit bark surface area	bark_dry_mass_per_surface_area	trait_0012014
Bark water content per unit bark dry mass	bark_water_content_per_dry_mass	trait_0012015
Bark water content per unit saturated bark mass	bark_water_content_per_saturated_mass	trait_0012016
Root diameter	root_diameter	trait_0012111
Root system morphology	root_system_classification	trait_0012112
Fine root volume to coarse root volume ratio	root_fine_root_coarse_root_ratio	trait_0012113
Root biomass depth distribution coefficient	root_distribution_coefficient	trait_0012114
Root system type (presence of taproot)	root_system_type	trait_0012115
Specific root length (SRL)	root_specific_root_length	trait_0012116
Specific tap root length (STRL)	root_specific_taproot_length	trait_0012117
Root surface area per unit root dry mass (specific root area)	root_specific_root_area	trait_0012118
Root wood density	root_wood_density	trait_0012119
Root to shoot ratio	root_shoot_ratio	trait_0012120
Root dry matter content (RDMC)	root_dry_matter_content	trait_0012121
Root mass fraction	root_mass_fraction	trait_0012122
Seed accessory cost fraction	accessory_cost_fraction	trait_0012221
Seed accessory cost mass	accessory_cost_mass	trait_0012222
Number of androecium parts in each whorl (Androecium structural merism)	flower_androecium_structural_merism	trait_0012410
Androecium structural phyllotaxis	flower_androecium_structural_phyllotaxis	trait_0012411
Number of androecium structural whorls	flower_androecium_structural_whorls_count	trait_0012412
Anther attachment	flower_anther_attachment	trait_0012413
Connective extension (apical)	flower_anther_connective_extension	trait_0012414
Anther dehiscence	flower_anther_dehiscence	trait_0012415
Anther orientation	flower_anther_orientation	trait_0012416
Flower colour	flower_colour	trait_0012417
Perianth colour	perianth_colour	trait_0012418
Flower length	flower_length	trait_0012419
Flower diameter	flower_diameter	trait_0012420
Floral orientation	flower_orientation	trait_0012421
Maximum flower number	flower_count_maximum	trait_0012422
Number of fertile stamens	flower_fertile_stamens_count	trait_0012431
Filament presence and shape	flower_filament	trait_0012432
Fusion of filaments	flower_filament_fusion	trait_0012433

Fusion of filaments to inner perianth series	flower_filament_fusion_to_inner_perianth	trait_0012434
Gynoecium phyllotaxis	flower_gynoecium_phyllotaxis	trait_0012441
Placentation	flower_gynoecium_placentation	trait_0012442
Fusion of ovaries	flower_ovary_fusion	trait_0012443
Ovary position	flower_ovary_position	trait_0012444
Number of ovules per functional carpel	flower_ovules_per_functional_carpel_count	trait_0012445
Perianth differentiation	flower_perianth_differentiation	trait_0012461
Fusion of perianth	flower_perianth_fusion	trait_0012462
Number of perianth parts in each whorl (Perianth merism)	flower_perianth_merism	trait_0012463
Number of perianth parts	flower_perianth_parts_count	trait_0012464
Perianth phyllotaxis	flower_perianth_phyllotaxis	trait_0012465
Symmetry of perianth	flower_perianth_symmetry	trait_0012466
Number of perianth whorls	flower_perianth_whorls_count	trait_0012467
Pollen grain aperture shape	flower_pollen_aperture_shape	trait_0012471
Number of pollen grain apertures	flower_pollen_apertures_count	trait_0012472
Pollen grain length	flower_pollen_length	trait_0012473
Number of structural carpels	flower_structural_carpels_count	trait_0012481
Floral structural sex	flower_structural_sex_type	trait_0012482
Style differentiation	flower_style_differentiation	trait_0012483
Fusion of styles	flower_style_fusion	trait_0012484
Fruit dry mass	fruit_dry_mass	trait_0012511
Fruit length	fruit_length	trait_0012512
Fruit width	fruit_width	trait_0012513
Fruit breadth	fruit_height	trait_0012514
Fruit wall thickness	fruit_wall_thickness	trait_0012515
Fruit type	fruit_type	trait_0012516
Fruit fleshiness	fruit_fleshiness	trait_0012517
Fruit dehiscence	fruit_dehiscence	trait_0012518
Fruit colour	fruit_colour	trait_0012519
Seed dry mass	seed_dry_mass	trait_0012610
Diaspore dry mass	diaspore_dry_mass	trait_0012611
Seed embryo and endosperm dry mass	seed_dry_mass_reserve	trait_0012612
Seed length	seed_length	trait_0012613
Seed width	seed_width	trait_0012614
Seed height	seed_height	trait_0012615
Seed volume	seed_volume	trait_0012616
Seed count	seed_count	trait_0012617
Seed shape	seed_shape	trait_0012618
Seed surface hairs	seed_surface_hairs	trait_0012619

Seed surface texture	seed_surface_texture	trait_0012620
Seed surface reflectivity	seed_surface_reflectivity	trait_0012621
Diaspore fleshiness	diaspore_fleshiness	trait_0012622
Dispersal appendage	dispersal_appendage	trait_0012623
Dispersal unit	dispersal_unit	trait_0012624
Leaf secondary vein angle	leaf_secondary_vein_angle	trait_0013011
Major leaf vein density	leaf_major_vein_density	trait_0013012
Length of all minor and major leaf lamina veins per unit area	leaf_total_vein_density	trait_0013013
Leaf vein frequency	leaf_vein_frequency	trait_0013014
Stem xylem vessel density	stem_vessel_density	trait_0013111
Leaf xylem vessel density	leaf_vessel_density	trait_0013112
Stem xylem vessel diameter	stem_vessel_diameter	trait_0013113
Stem xylem vessel hydraulic mean diameter	stem_vessel_diameter_hydraulic	trait_0013114
Leaf xylem vessel diameter	leaf_vessel_diameter	trait_0013115
Stem xylem vessel lumen fraction	stem_vessel_lumen_fraction	trait_0013116
Stem xylem vessel multiple fraction	stem_vessel_multiple_fraction	trait_0013117
Stem non-lumen fraction	stem_vessel_non_lumen_fraction	trait_0013118
Xylem vessel wall fraction	stem_vessel_wall_fraction	trait_0013119
Xylem vulnerability index	stem_xylem_vulnerability_index	trait_0013120
Wood axial parenchyma fraction	wood_axial_parenchyma_fraction	trait_0013161
Wood conduit fraction	wood_conduit_fraction	trait_0013162
Wood fibre fraction	wood_fibre_fraction	trait_0013163
Wood ray parenchyma fraction	wood_ray_parenchyma_fraction	trait_0013164
Wood tracheid fraction	wood_tracheid_fraction	trait_0013165
Leaf work to punch	leaf_work_to_punch	trait_0014011
Leaf specific work to punch	leaf_work_to_punch_adjusted	trait_0014012
Leaf work to shear	leaf_work_to_shear	trait_0014013
Leaf specific work to shear (fracture toughness)	leaf_work_to_shear_adjusted	trait_0014014
Leaf work to tear	leaf_work_to_tear	trait_0014015
Leaf specific work to tear	leaf_work_to_tear_adjusted	trait_0014016
Bark modulus of elasticity	bark_modulus_of_elasticity	trait_0014017
Stem modulus of elasticity	stem_modulus_of_elasticity	trait_0014018
Xylem modulus of elasticity	xylem_modulus_of_elasticity	trait_0014019
Modulus of rupture	modulus_of_rupture	trait_0014020
**Biological Process Trait (Physiological Process Trait)**		
Plant photosynthetic pathway	photosynthetic_pathway	trait_0020221

Bark photosynthesis	bark_photosynthetic_status	trait_0020222
Leaf photosynthesis rate per unit leaf area under ambient light and CO2 (A)	leaf_photosynthetic_rate_per_area_ambient	trait_0020240
Leaf photosynthesis rate per unit leaf area under saturating light and CO2 (Amax)	leaf_photosynthetic_rate_per_area_maximum	trait_0020241
Leaf photosynthesis rate per unit leaf area under saturating light and ambient CO2 (Asat)	leaf_photosynthetic_rate_per_area_saturated	trait_0020242
Leaf photosynthesis rate per unit leaf dry mass under ambient light and CO2 (A)	leaf_photosynthetic_rate_per_dry_mass_ambient	trait_0020243
Leaf photosynthesis rate per unit leaf dry mass under saturating light and CO2 (Amax)	leaf_photosynthetic_rate_per_dry_mass_maximum	trait_0020244
Leaf photosynthesis rate per unit leaf dry mass under saturating light and ambient CO2 (Asat)	leaf_photosynthetic_rate_per_dry_mass_saturated	trait_0020245
Leaf internal CO2 concentration during Amax measurement (ci)	leaf_intercellular_CO2_concentration_at_Amax	trait_0020310
Internal CO2 concentration during Asat measurement (ci)	leaf_intercellular_CO2_concentration_at_Asat	trait_0020311
Internal CO2 concentration under ambient conditions (ci)	leaf_intercellular_CO2_concentration_at_Aambient	trait_0020312
Ratio of internal to external CO2 concentrations (ci/ca)	leaf_intercellular_CO2_concentration_to_atmospheric_CO2_concentration_ratio	trait_0020313
CO2 concentration inside chloroplasts (cc)	leaf_chloroplast_CO2_concentration	trait_0020314
Ambient CO2 concentration (ca)	atmospheric_CO2_concentration	trait_0020315
Leaf Jmax per unit leaf area (Jmax)	leaf_photosynthesis_Jmax_per_area	trait_0020410
Leaf Jmax per unit leaf area at 25 deg C (Jmax25)	leaf_photosynthesis_Jmax_per_area_25C	trait_0020411
Leaf Jmax per unit leaf mass (Jmax)	leaf_photosynthesis_Jmax_per_mass	trait_0020412
Leaf Vcmax per unit leaf area (Vcmax)	leaf_photosynthesis_Vcmax_per_area	trait_0020413
Leaf Vcmax per unit leaf area at 25 deg C (Vcmax25)	leaf_photosynthesis_Vcmax_per_area_25C	trait_0020414
Leaf Vcmax per unit leaf mass (Vcmax)	leaf_photosynthesis_Vcmax_per_mass	trait_0020415
Leaf Jmax to leaf Vcmax ratio at 25 deg C	leaf_photosynthesis_Jmax_over_Vcmax_25C	trait_0020416
Leaf maximum quantum yield (Fv/Fm)	leaf_fluorescence_fv_over_fm	trait_0020417
Leaf ambient quantum yield	leaf_fluorescence_quantum_yield	trait_0020418
Leaf quantum yield, gas exchange measurement	leaf_gas_exchange_quantum_yield	trait_0020419

Leaf respiration rate per unit leaf area, in the dark (Rdark)	leaf_dark_respiration_per_area	trait_0020510
Leaf respiration rate per unit leaf dry mass, in the dark (Rdark)	leaf_dark_respiration_per_dry_mass	trait_0020511
Leaf respiration rate per unit leaf area, in the light (Rday)	leaf_light_respiration_per_area	trait_0020512
Stem respiration rate per unit stem area, in the dark	stem_dark_respiration_per_area	trait_0020513
Leaf stomatal conductance to water vapour per unit leaf area under ambient conditions (gsw)	leaf_stomatal_conductance_per_area_ambient	trait_0020610
Leaf stomatal conductance to water vapour per unit leaf area during Amax measurement (gsw)	leaf_stomatal_conductance_per_area_at_Ama x	trait_0020611
Leaf stomatal conductance to water vapour per unit leaf area during Asat measurement (gsw)	leaf_stomatal_conductance_per_area_at_Asat	trait_0020612
Leaf stomatal water vapour resistance under ambient conditions	leaf_stomatal_resistance_ambient	trait_0020630
Leaf mesophyll conductance to carbon dioxide per unit leaf area (gm)	leaf_mesophyll_conductance_per_area	trait_0020640
Leaf mesophyll conductance to carbon dioxide per unit leaf mass (gm)	leaf_mesophyll_conductance_per_mass	trait_0020641
Leaf transpiration per unit leaf area under ambient conditions (E)	leaf_transpiration_per_area_ambient	trait_0020660
Leaf transpiration per unit leaf area during Amax measurement (E)	leaf_transpiration_per_area_at_Amax	trait_0020661
Leaf transpiration per unit leaf area during Asat measurement (E)	leaf_transpiration_per_area_at_Asat	trait_0020662
Leaf transpiration rate per unit leaf area, in the dark	leaf_dark_transpiration_per_area	trait_0020663
Integrated plant transpiration	integrated_plant_transpiration	trait_0020664
Whole plant sapflow	whole_plant_sapflow	trait_0020665
Leaf photosynthetic nitrogen use efficiency during Amax measurement (PNUE)	leaf_photosynthetic_nitrogen_use_efficiency_ maximum	trait_0020710
Leaf photosynthetic nitrogen use efficiency during Asat measurement (PNUE)	leaf_photosynthetic_nitrogen_use_efficiency_s aturated	trait_0020711
Leaf photosynthetic phosphorus use efficiency during Amax measurement PPUE)	leaf_photosynthetic_phosphorus_use_efficienc y_maximum	trait_0020712

Leaf photosynthetic phosphorus use efficiency during Asat measurement (PPUE)	leaf_photosynthetic_phosphorus_use_efficiency_saturated	trait_0020713
Integrated water use efficiency	leaf_water_use_efficiency_integrated	trait_0020760
Intrinsic water use efficiency (WUEi)	leaf_water_use_efficiency_intrinsic	trait_0020761
Instantaneous water use efficiency (WUE)	leaf_water_use_efficiency_instantaneous	trait_0020762
Stem hydraulic conductivity (Kh)	stem_hydraulic_conductivity	trait_0021013
Sapwood specific hydraulic conductivity (Ks)	sapwood_specific_hydraulic_conductivity	trait_0021014
Theoretical sapwood specific hydraulic conductivity (Ks)	sapwood_specific_hydraulic_conductivity_theoretical	trait_0021015
Stem specific hydraulic conductivity (Ks)	stem_specific_hydraulic_conductivity	trait_0021016
Leaf specific hydraulic conductance (kleaf)	leaf_specific_hydraulic_conductance	trait_0021017
Leaf specific hydraulic conductivity (Kl)	leaf_specific_hydraulic_conductivity	trait_0021018
Root hydraulic conductivity (Kh)	root_hydraulic_conductivity	trait_0021021
Root specific hydraulic conductivity	root_specific_hydraulic_conductivity	trait_0021022
Pre-dawn water potential	water_potential_predawn	trait_0021030
Midday water potential	water_potential_midday	trait_0021031
Stem sapwood capacitance (C)	stem_sapwood_capacitance	trait_0021032
Leaf capacitance (Cleaf)	leaf_capacitance	trait_0021033
Root sapwood capacitance (C)	root_sapwood_capacitance	trait_0021034
Leaf xylem pressure, 50% lost conductance	leaf_hydraulic_vulnerability	trait_0021050
Stem xylem pressure, 12% lost conductivity	water_potential_12percent_lost_conductivity	trait_0021051
Stem xylem pressure, 50% lost conductivity	water_potential_50percent_lost_conductivity	trait_0021052
Stem xylem pressure, 88% lost conductivity	water_potential_88percent_lost_conductivity	trait_0021053
Hydraulic safety margin, 50%	hydraulic_safety_margin_50	trait_0021054
Hydraulic safety margin, 88%	hydraulic_safety_margin_88	trait_0021055
Leaf turgor loss point	leaf_turgor_loss_point	trait_0021056
Osmotic potential	osmotic_potential	trait_0021057
Osmotic potential at full tugor	osmotic_potential_at_full_turgor	trait_0021058
Bulk modulus of elasticity (e)	bulk_modulus_of_elasticity	trait_0021059
Leaf relative water content predawn	leaf_relative_water_content_predawn	trait_0021060
Leaf relative water content at turgor loss point	leaf_relative_water_content_at_turgor_loss_point	trait_0021061
Root xylem pressure, 50% lost conductivity	root_water_potential_50percent_lost_conductivity	trait_0021062
Root xylem pressure, 12% lost conductivity (P12)	root_water_potential_12percent_lost_conductivity	trait_0021063
Root xylem pressure, 88% lost conductivity (P88)	root_water_potential_88percent_lost_conductivity	trait_0021064

Leaf xylem pressure, 12% lost conductance (P12)	leaf_water_potential_12percent_lost_conductivity	trait_0021065
Leaf xylem pressure, 88% lost conductance (P88)	leaf_water_potential_88percent_lost_conductivity	trait_0021066
Photochemical reflectance index (PRI)	leaf_photochemical_reflectance_index	trait_0020813
Water band index	leaf_water_band_index	trait_0020814
Modified normalized difference vegetation index (modified NDVI)	modified_NDVI	trait_0020815
Modified chlorophyll absorption ratio index 705	leaf_chlorophyll_index_modified_ND705	trait_0020816
**Life History Trait**		
Plant growth form	plant_growth_form	trait_0030010
Life form	life_form	trait_0030011
Life history	life_history	trait_0030012
Ephemeral life history class	life_history_ephemeral_class	trait_0030013
Lifespan	lifespan	trait_0030014
Plant growth substrate	plant_growth_substrate	trait_0030015
Plant photosynthetic organ	plant_photosynthetic_organ	trait_0030016
Plant alternative energy and nutrient acquisition strategies	plant_alternative_energy_and_nutrient_acquisition_strategy	trait_0030017
Woodiness	woodiness	trait_0030018
Detailed woodiness categories	woodiness_detailed	trait_0030019
Physical defence structures	plant_physical_defence_structures	trait_0030020
Plant climbing mechanisms	plant_climbing_mechanism	trait_0030021
Plant succulence	plant_succulence	trait_0030022
Stem growth habit	stem_growth_habit	trait_0030023
Leaf phenology	leaf_phenology	trait_0030024
Leaf lifespan	leaf_lifespan	trait_0030025
Competitive stratum	competitive_stratum	trait_0030026
Plant nitrogen fixation capacity	nitrogen_fixing	trait_0030027
Plant root structures	root_structure	trait_0030028
Plant parasitism status	parasitic	trait_0030029
Plant sex type	sex_type	trait_0030060
Pollination syndrome	pollination_syndrome	trait_0030061
Pollination system	pollination_system	trait_0030062
Plant genome size	genome_size	trait_0030080
Chromosome ploidy	ploidy	trait_0030081
Age of reproductive maturity	reproductive_maturity	trait_0030210
Diaspore dispersal syndrome	dispersal_syndrome	trait_0030211
Diaspore dispersal agents	dispersers	trait_0030212

Environmental flowering cues	flowering_cues	trait_0030213
Flowering time, by month	flowering_time	trait_0030214
Fruiting time, by month	fruiting_time	trait_0030215
Seedling recruitment time, by month	recruitment_time	trait_0030216
Seedling establishment conditions	seedling_establishment_conditions	trait_0030217
Canopy light environment required for reproduction	reproductive_light_environment_index	trait_0030218
Canopy light environment required for seedling establishment	establishment_light_environment_index	trait_0030219
Seed storage location	seedbank_location	trait_0030411
Serotiny	serotiny	trait_0030412
Seedbank longevity class	seedbank_longevity_class	trait_0030413
Seedbank longevity	seedbank_longevity	trait_0030414
Dormancy type	seed_dormancy_class	trait_0030415
Seed germination treatment	seed_germination_treatment	trait_0030416
Seed germination proportion	seed_germination	trait_0030417
Seed viability	seed_viability	trait_0030418
Seed germination time	seed_germination_time	trait_0030419
Vegetative reproduction ability	vegetative_reproduction_ability	trait_0030510
Clonal spread mechanism	clonal_spread_mechanism	trait_0030511
Storage organ	storage_organ	trait_0030512
Bud bank location	bud_bank_location	trait_0030513
Sprout depth	sprout_depth	trait_0030514
Post-fire resprouting capacity	resprouting_capacity	trait_0030610
Post-fire proportion resprouting individuals	resprouting_capacity_proportion_individuals	trait_0030611
Post-fire resprouting capacity of juvenile plants	resprouting_capacity_juvenile	trait_0030612
Time from seedling germination until individuals survive a fire	resprouting_capacity_time_from_germination	trait_0030613
Post-fire to pre-fire stem ratio	resprouting_capacity_stem_ratio	trait_0030614
Plant vegetative response to disturbances other than fire	resprouting_capacity_non_fire_disturbance	trait_0030615
Fire exposure level	fire_exposure_level	trait_0030651
Post-fire recruitment	post_fire_recruitment	trait_0030652
Post-fire flowering	post_fire_flowering	trait_0030653
Time from fire to first flowering	fire_time_from_fire_to_flowering	trait_0030654
Time from fire until 50% of individuals are flowering	fire_time_from_fire_to_50_percent_flowering	trait_0030655
Time from fire to peak flowering	fire_time_from_fire_to_peak_flowering	trait_0030656
Time from fire until flowering declines	fire_time_from_fire_to_flowering_decline	trait_0030657

Time from fire to fruiting	fire_time_from_fire_to_fruiting	trait_0030658
Time from fire until 50% of individuals are fruiting	fire_time_from_fire_to_50_percent_fruiting	trait_0030659
Fuel bed bulk density	fire_fuel_bed_bulk_density	trait_0030710
Fuel consumption by fire	fire_fuel_consumption	trait_0030711
Fire rate of spread	fire_rate_of_spread	trait_0030712
Leaf smoulder duration	fire_smoulder_duration	trait_0030713
Leaf flame duration	fire_flame_duration	trait_0030714
Leaf flame and smoulder duration	fire_total_burn_duration	trait_0030715
Fire time to ignition	fire_time_to_ignition	trait_0030716
Plant resource requirements and tolerance	plant_type_by_resource_use	trait_0030810
Plant flood regime response	plant_flood_regime_classification	trait_0030811
Plant water-logging tolerance	plant_tolerance_water_logged_soils	trait_0030812
Plant inundation tolerance	plant_tolerance_inundation	trait_0030813
Plant snow tolerance	plant_tolerance_snow	trait_0030814
Plant soil salinity tolerance	plant_tolerance_soil_salinity	trait_0030815
Plant salt tolerance strategy	plant_tolerance_salt	trait_0030816
Plant calcium sensitivity	plant_tolerance_calcicole	trait_0030817
Plant fire tolerance strategy	plant_tolerance_fire	trait_0030818

Table S14. Published vocabularies from which classes (terms) were sourced as metadata for a property within the APD.

**Vocabulary**	**Abbreviation**	**Scheme**
Cell Ontology	CL	http://purl.obolibrary.org/obo/
Cerrado	Cerrado	http://cerrado.linkeddata.es/ecology/
Chemical Entities of Biological Interest	CHEBI	http://purl.obolibrary.org/obo/
Chemical Methods Ontology	CHMO	http://purl.obolibrary.org/obo/
Common Anatomy Reference Ontology	CARO	http://purl.obolibrary.org/obo/
CorVeg	CorVeg	http://linked.data.gov.au/def/corveg-cv/
DICOM Controlled Terminology	DCM	http://dicom.nema.org/resources/ontology/DCM/
Ecocore Ontology	ECOCORE	http://purl.obolibrary.org/obo/
Ecosystem Ontology	ENVO	http://purl.dataone.org/odo/
Edam - Bioscientific Analysis Ontology	EDAM	http://edamontology.org/
Environment Ontology	ENVO	http://purl.obolibrary.org/obo/
Environmental Thesaurus	EnvThes	http://vocabs.lter-europe.net/EnvThes/
Experimental Conditions Ontology	XCO	http://purl.obolibrary.org/obo/
Experimental Factor Ontology	EFO	http://www.ebi.ac.uk/efo/
Flora Phenotype Ontology	FLOPO	http://purl.obolibrary.org/obo/
Gene Ontology	GO	http://purl.obolibrary.org/obo/
Human Phenotype Ontology	HP	http://purl.obolibrary.org/obo/
Human Physiology Simulation Ontology	hupson	http://scai.fraunhofer.de/HuPSON#
Interlinking Ontology for Biological Concepts	IOBC	http://purl.jp/bio/4/id/
Invasion Biology Ontology	INBIO	http://ncicb.nci.nih.gov/xml/owl/EVS/Thesaurus.owl#
Medical Subject Headings	MESH	http://purl.bioontology.org/ontology/MESH/
Molecular Process Ontology	MOP	http://purl.obolibrary.org/obo/
MOSAiC Ontology	MOSAIC	http://purl.dataone.org/odo/
National Cancer Institute Thesaurus	NCIT	http://purl.obolibrary.org/obo/
NCBI organismal classification	NCBITaxon	http://purl.obolibrary.org/obo/
Ontology for Biomedical Investigations	OBI	http://purl.obolibrary.org/obo/

Ontology for MicroRNA Target	OMIT	http://purl.obolibrary.org/obo/
Ontology of units of Measure	om	http://www.ontology-of-units-of-measure.org/resource/om-2/
Phenotypic Quality Ontology	PATO	http://purl.obolibrary.org/obo/
Plant Ontology	PO	http://purl.obolibrary.org/obo/
Plant Trait Ontology	TO	http://purl.obolibrary.org/obo/
Population and Community Ontology	PCO	http://purl.obolibrary.org/obo/
Semantic Web for Earth and Environment Technology Ontology	SWEET	http://sweetontology.net/
Semanticscience Integrated Ontology	SIO	http://semanticscience.org/resource/
Solanaceae Phenotype Ontology [SPTO]	SP	http://purl.obolibrary.org/obo/
Statistical Methods Ontology	STATO	http://purl.obolibrary.org/obo/
Uber Anatomy Ontology (UBERON)	UBERON	http://purl.obolibrary.org/obo/
Units of measurement ontology	UO	http://purl.obolibrary.org/obo/
Wheat Trait Ontology	CO_321	https://cropontology.org/rdf/
Woody Plant Ontology	CO_357	https://cropontology.org/rdf/

Table S15. Columns in the data table APD_triples.csv

Column	Description
Subject	the URI for a concept (class)
property	English label for the Predicate
Predicate	a URI for a property
value	English label or string that is the value of a specific property (Predicate) for a specific concept (Subject)
Object	a URI for an object, only filled in when the Object has a URI not a string
graph	the graph the Subject, Predicate, Object triple is part of in a dataset; required for .nq output, filled as \`.\` (blank) throughout the APD

Table S16. Output for the trait `life history` from APD.ttl

Sample output of machine readable code for a single trait, extracted from the data file APD.ttl.

```
APD:trait_0030012
    a owl:Class, skos:Concept ;
    rdfs:label "Life history"@en ;
    skos:prefLabel "Life history"@en ;
    skos:altLabel "life_history" ;
    skos:definition "Categorical description of the duration [PATO:0001309] of a plant's lifespan (longevity
[NCIT:C153298]), from seed germination [GO:0009845] to death [GO:0016265]."@en, "Categorical
description of the duration of a plant's lifespan, from germination to death."@en ;
    dcterms:description "Categorical description of the duration [PATO:0001309] of a plant's lifespan
(longevity [NCIT:C153298]), from seed germination [GO:0009845] to death [GO:0016265]."@en,
"Categorical description of the duration of a plant's lifespan, from germination to death."@en ;
    rdfs:comment "Studies will differ in the subset of terms they use to describe a plant's life history, such
that some researchers will distinguish between ephemeral and annual species, and other researchers
will group these life history categories together under `annual`. In addition, only a subset of studies will
use the term `short-lived perennial`; the majority will score all perennial plants as `perennial`. Rangeland
studies and post-fire studies are those most likely to score species as `ephemeral` or `short-lived
perennial`, as these are environments where perennial species' lifespans are often divided into those
that are short-lived due to environmental conditions and those that are able to persist through the
environmentally unfavourable period."@en ;
    dcterms:identifier "trait_0030012" ;
    ets:valueType obo:STATO_0000252 ;
    oboecore:MeasuredCharacteristic obo:PATO_0000165, obo:PATO_0001309, obo:PATO_0001995 ;
    ont:hasContextObject obo:PO_0000003 ;
    skos:narrower APD:life_history_annual, APD:life_history_biennial, APD:life_history_ephemeral,
APD:life_history_perennial, APD:life_history_short_lived_perennial ;
    datacite:IsReviewedBy <https://orcid.org/0000-0001-5640-5910>, <https://orcid.org/0000-0001-
8305-3236>, <https://orcid.org/0000-0001-8338-9143>, <https://orcid.org/0000-0002-0712-5143>,
<https://orcid.org/0000-0002-1773-6597>, <https://orcid.org/0000-0002-6033-2766>,
<https://orcid.org/0000-0003-0360-8321>, <https://orcid.org/0000-0003-1116-9402>,
<https://orcid.org/0000-0003-2008-7062>, <https://orcid.org/0000-0003-3568-2606> ;
    dcterms:created "14/07/2021"^^<xsd:date> ;
   dcterms:modified "05/02/2024"^^<xsd:date> ;
    dcterms:reviewed "31/10/2022"^^<xsd:date> ;
    dcterms:references <https://doi.org/10.1071/BT12225>,
<https://uol.de/en/landeco/research/leda/standards> ;
    SIO:SIO_000147 obo:GO_0016265, obo:NCIT_C153298 ;
    rdfs:subClassOf APD:trait_group_0030006 ;
    skos:broader APD:trait_group_0030006 ;
    skos:example "close match: plant lifespan and age of first flowering [LEDA:1.3] (https://www.try-
db.org/de/de.php)" ; " close match: plant lifespan and age of first flowering [LEDA:1.3]
```

(https://www.try-db.org/de/de.php)" ; "exact match: Plant lifespan (longevity) [TRY:59] (https://www.try-db.org/de/de.php)", "exact match: lifecycle [GIFT:2.1.1] (https://gift.uni-goettingen.de)"; "related match: Growth form [BROT:1] (http://doi.org/10.1038/sdata.2018.135) (http://doi.org/10.1038/sdata.2018.135)" ;
    skos:exactMatch obo:TO_0002725 ;
    skos:scopeNote "none"@en ;
    skos:inScheme "https://w3id.org/APD/traits" .
